# metaSEM: an R package for meta-analysis using structural equation modeling

**DOI:** 10.3389/fpsyg.2014.01521

**Published:** 2015-01-05

**Authors:** Mike W.-L. Cheung

**Affiliations:** Department of Psychology, National University of SingaporeSingapore, Singapore

**Keywords:** meta-analysis, structural equation modeling, meta-analytic structural equation modeling, metaSEM, R

## Abstract

The metaSEM package provides functions to conduct univariate, multivariate, and three-level meta-analyses using a structural equation modeling (SEM) approach via the OpenMx package in the R statistical platform. It also implements the two-stage SEM approach to conducting fixed- and random-effects meta-analytic SEM on correlation or covariance matrices. This paper briefly outlines the theories and their implementations. It provides a summary on how meta-analyses can be formulated as structural equation models. The paper closes with a conclusion on several relevant topics to this SEM-based meta-analysis. Several examples are used to illustrate the procedures in the supplementary material.

## 1. Introduction

Meta-analysis is a popular technique for synthesizing research findings in the social, behavioral, educational, and medical sciences (e.g., Hedges and Olkin, 1985; Whitehead, 2002; Borenstein et al., 2009; Schmidt and Hunter, 2015). There are several standalone programs for conducting meta-analyses, e.g., Comprehensive Meta-Analysis (Borenstein et al., 2005). There are also macros or packages to fit some meta-analytic models in standard statistical packages such as SPSS (Lipsey and Wilson, 2000), and SAS (Arthur et al., 2001). R (R Development Core Team, 2014) is a popular open source statistical platform for computations and data analysis. There are also several R packages available for meta-analysis (e.g., Viechtbauer, 2010; Lumley, 2012; Schwarzer, 2014).

The metaSEM package (Cheung, 2014b) is another R package for conducting meta-analyses. It formulates univariate, multivariate, and three-level meta-analytic models as structural equation models (Cheung, 2008, 2013b, 2014c, in press) via the OpenMx package (Boker et al., 2011). It also implements the two-stage structural equation modeling (TSSEM) approach (Cheung and Chan, 2005, 2009; Cheung, 2014a) to fit fixed- and random-effects meta-analytic structural equation modeling (MASEM) on correlation or covariance matrices. This paper outlines the meta-analytic models implemented in the metaSEM package (Cheung, in press). There are two main objectives of this paper. First, it provides an succinct summary on how various meta-analytic models can be formulated as structural equation models. Readers may refer to the references for more details and advantages of formulating meta-analytic models as structural equation models. Second, it illustrates how to conduct these analyses using the metaSEM package. Complete R code, output, and remarks are included in the supplementary material. Users may refer to http://courses.nus.edu.sg/course/psycwlm/Internet/metaSEM/ on how to install the metaSEM package.

## 2. Structural equation modeling based meta-analysis

SEM is a multivariate technique to fit and test hypothesized models. Let **y** be a *p* × 1 vector of a sample of continuous data from a multivariate normal distribution where *p* is the number of observed variables. It is hypothesized that the model for the first and the second moments are functions of θ, where θ is a vector of parameters that can be regression coefficients, error variances, factor loadings, and factor variances. The model is:

(1) $$\begin{array}{l} \mu =\mu (\theta )\text{ and}\\ \Sigma =\Sigma (\theta ), \end{array}$$

where μ and Σ are the population mean vector and covariance matrix, respectively. Maximum likelihood (ML) estimation method is the most common estimation method in SEM. The −2^*^log-likelihood (−2*LL*) for the *i*th case is,

(2) $$\begin{array}{l} -2LL_{i}{\left(\theta ;y_{i}\right)}_{\text{ML}}=p_{i}\log\left(2\pi \right)+\log|\Sigma_{i}\left(\theta \right)|\\ \;+\;{\left(y_{i}-\mu_{i}\left(\theta \right)\right)}^{⊤}\Sigma_{i}{\left(\theta \right)}^{-1}\left(y_{i}-\mu_{i}\left(\theta \right)\right), \end{array}$$

where *p*_*i*_ is the number of filtered variables with complete data in the *i*th case, μ_*i*_(θ) and Σ_*i*_(θ) are the model implied mean vector and the model implied covariance matrix for the *i*th case, respectively. Since there is a subscript *i* in Equation 2, the model implied mean vector and covariance matrix may vary across cases. Thus, it automatically handles incomplete data by selecting the complete data in the log-likelihood function with the full information maximum likelihood (ML or FIML) estimation method (Enders, 2010).

To obtain the parameter estimates, we may take the sum of the −2*LL*_*i*_ over all cases and minimize it. Iterative methods are used to obtain the parameter estimates. When it is convergent, the asymptotic sampling covariance matrix of the parameter estimates may be obtained from the inverse of the Hessian matrix. The standard errors (*SE*s) of the parameter estimates are calculated by taking the square root of the diagonal elements of the asymptotic sampling covariance matrix. The parameter estimates divided by their *SE*s follow a *z* distribution under the null hypothesis. A likelihood ratio (*LR*) statistic may also be used to compare nested models. The model fit and the significance of individual parameters can be tested (e.g., Kline, 2011).

### 2.1. Univariate fixed-effects model

The following subsections briefly introduce how various meta-analytic models can be formulated as structural equation models. Let us begin with the meta-analytic model with only one effect size *y*_*i*_ in the *i*th study (Cheung, 2008). *y*_*i*_ can be any effect size, such as the odds ratio, raw mean difference, standardized mean difference, correlation coefficient, or its Fisher's z transformed score. When the sample sizes in the primary studies are reasonably large, *y*_*i*_ can be assumed to be normally distributed with a variance of *v*_*i*_ (e.g., see Borenstein et al., 2009, for the formulas of common effect sizes. The univariate fixed-effects model for the *i*th study is:

(3) $$y_{i}=\beta_{\text{F}}+e_{i},$$

where β_*F*_ is the common effect under the fixed-effects model, and Var(*e*_*i*_) = *v*_*i*_ is the known sampling variance. To conduct a univariate fixed-effects meta-analysis in SEM, we may fit the following model implied moments:

(4) $$\begin{array}{l} \mu_{i}(\theta )=\beta_{\text{F}}\text{ and}\\ \Sigma_{i}(\theta )=v_{i}. \end{array}$$

**Figure 1:**
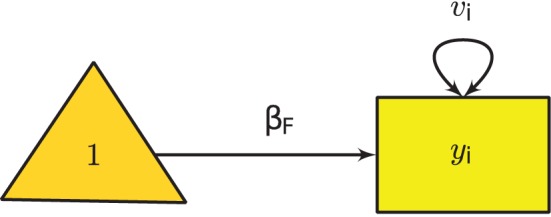
**Univariate fixed-effects meta-analysis**.

Since *v*_*i*_ is known, the only parameter in the model is β_*F*_. Figure 1 shows the graphical model of the fixed-effects meta-analysis.

### 2.2. Univariate random-effects model

Since the primary studies are conducted by different researchers in different settings, these studies are unlikely not direct replicates of each other. It is reasonable to expect that the population effect sizes may not be the same. A random-effects model allows studies to have their own study-specific effect. The model for the *i*th study is:

(5) $$y_{i}=\beta_{\text{R}}+u_{i}+e_{i},$$

where β_R_ is the average population effect under the random-effects model, and Var(*u*_*i*_) = τ^2^ is the heterogeneity variance that has to be estimated. To fit the model in SEM, we may consider the following model implied moments:

(6) $$\begin{array}{l} \mu_{i}(\theta )=\beta_{\text{R}}\text{ and}\\ \Sigma_{i}(\theta )=\tau^{2}+v_{i}. \end{array}$$

**Figure 2:**
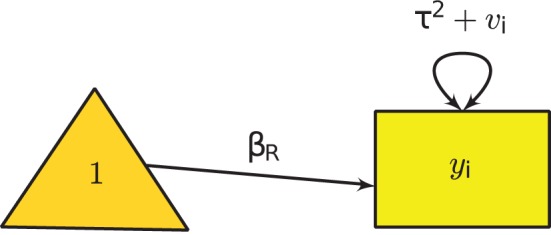
**Univariate random-effects meta-analysis**.

In the literature of meta-analysis, *v*_*i*_ and τ^2^ + *v*_*i*_ are known as the conditional and the unconditional variances, respectively. Under this model we have to estimate both β_R_ and τ^2^. Figure 2 shows the graphical model of the random-effects meta-analysis. Various estimation methods, such as methods of moments, ML estimation and restricted maximum likelihood (REML) estimation may be used to estimate τ^2^ (e.g., Borenstein et al., 2009). The default estimation method in the SEM-based meta-analysis is ML estimation, while the REML estimation method may also be used to minimize the slight negative bias on the estimated variance component using the ML estimation method (Cheung, 2013a).

#### 2.2.1. Quantifying heterogeneity

To test the homogeneity of the population effect sizes, we may compute the *Q* statistic (Cochran, 1954),

(7) $$Q=\sum_{i\;=\;1}^{k}w_{i}{\left(y_{i}-{\hat{\beta }}_{\text{F}}\right)}^{2},$$

where *w*_*i*_ = 1/*v*_*i*_. Under the null hypothesis of the homogeneity of effect sizes, the *Q* statistic has an approximate chi-square distribution with (*k* − 1) degrees of freedom (*df*s). The *Q* statistic may be significant simply because of the large number of studies. Conversely, a large *Q* statistic may be non-significant because of the small number of studies. Therefore, the significance of the *Q* statistic should not be used to determine whether a fixed- or a random-effects model is used in the analysis.

One popular index quantifying the degree of heterogeneity of effect sizes is the *I*^2^ (Higgins and Thompson, 2002). The general formula is

(8) $$I^{2}=\frac{{\hat{\tau }}^{2}}{{\hat{\tau }}^{2}+\tilde{v}},$$

where $\tilde{v}$ is a *typical* within-study sampling variance. *I*^2^ can be interpreted as the proportion of the total variation of the effect size that is due to the between study heterogeneity. Higgins and Thompson (2002) defined the *typical* within-study sampling variance using the *Q* statistic:

(9) $${\tilde{v}}_{\text{Q}}=\frac{(k-1)\sum_{i\;=\;1}^{k}1/v_{i}}{{\left(\sum_{i\;=\;1}^{k}1/v_{i}\right)}^{2}-\sum_{i\;=\;1}^{k}1/v_{i}^{2}}.$$

One advantage of using $\tilde{v}$_*Q*_ as the *typical* within-study sampling variance is that *I*^2^ can be simplified to *I*^2^_*Q*_ = *Q* − (*k* − 1)/*Q*.

Two more definitions of $\tilde{v}$ have also been proposed in the literature. Takkouche et al. (1999) suggested that the harmonic mean of *v*_*i*_ can be used as the *typical* within-study sampling variance,

(10) $${\tilde{v}}_{\text{HM}}=\frac{k}{\sum_{i\;=\;1}^{k}1/v_{i}}.$$

Xiong et al. (2010) also discussed an estimator of *I*^2^ that is based on the arithmetic mean:

(11) $${\tilde{v}}_{\text{AM}}=\sum_{i\;=\;1}^{k}v_{i}/k.$$

All of the above definitions are available in the metaSEM package. Users may choose among them by specifying the argument I2 = “I2q” based on the *Q* statistic (the default), I2 = “I2hm” based on the harmonic mean, and I2 = “I2am” based on the arithmetic mean.

### 2.3. Univariate mixed-effects model

The mixed-effects meta-analysis extends the random-effects meta-analysis by using study characteristics as predictors. Assuming that **x**_*i*_ is an (*m* + 1) × 1 vector of predictors including a constant of 1 where *m* is the number predictors in the *i*th study, the mixed-effects model is:

(12) $$y_{i}=x_{i}^{⊤}\beta +u_{i}+e_{i},$$

where β is a a (*m* + 1) × 1 vector of regression coefficients including the intercept. To fit the model in SEM, we may use the following model implied conditional mean and variance:

(13) $$\begin{array}{l} \mu_{i}(\theta |x_{i})=x_{i}^{⊤}\beta \text{ and}\\ \Sigma_{i}(\theta |x_{i})=\tau^{2}+v_{i}. \end{array}$$

**Figure 3:**
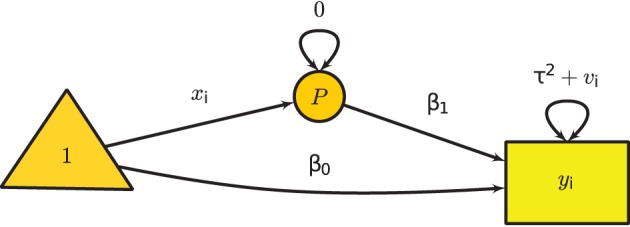
**Univariate mixed-effects meta-analysis with one predictor**.

Figure 3 shows the graphical model of the mixed-effects meta-analysis with one predictor. A phantom variable *P* is introduced to specify the predictor **x**_*i*_. Since **x**_*i*_ is specified via definition variables (see Cheung, 2010), **x**_*i*_ is treated as a design matrix rather than as variables.

Mathematically, it is clear that the random-effects meta-analysis is a special case of the mixed-effects meta-analysis by fixing **x** = **1** as a constant of ones, while the fixed-effects meta-analysis is a special case of the random-effects meta-analysis by fixing τ^2^ = 0. It should be noted that the assumptions and interpretations on the fixed- and random-effects models are different.

#### 2.3.1. Explained variance

Besides testing whether the predictors are significant, researchers may want to quantify the degree of prediction. The percentage of variance explained by the inclusion of predictors,

(14) $$R^{2}=\frac{{\hat{\tau }}_{0}^{2}-{\hat{\tau }}_{1}^{2}}{{\hat{\tau }}_{0}^{2}},$$

can be calculated by comparing the $\hat{\tau }$^2^_0_ without a predictor and the $\hat{\tau }$^2^_1_ with predictors (Raudenbush, 2009). When the calculated *R*^2^ is negative, it is usually truncated to zero.

### 2.4. Multivariate meta-analysis

When the research questions become more complicated, a single effect size may not be sufficient to summarize the effect in the primary studies. Multiple effect sizes are required to quantify the effect of the studies. Let us assume that there are a total of *p* effect sizes with *m* predictors in *k* studies. Since it is likely that different numbers of effect sizes are reported in the primary studies, we assume that there are *p*_*i*_ effect sizes in the *i*th study. The model for the multivariate mixed-effects meta-analysis in the *i*th study is:

(15) $$y_{i}=B_{i}x_{i}+Z_{i}u_{i}+e_{i},$$

where **y**_*i*_ is a *p*_*i*_ × 1 vector of effect sizes, **B**_*i*_ is a *p*_*i*_ × (*m* + 1) matrix of regression coefficients including the intercepts, **x**_*i*_ is a (*m* + 1) × 1 matrix of predictors including 1 in the first column, **Z**_*i*_ is a *p*_*i*_ × *p* filter matrix selecting the effect sizes that are present, **u**_*i*_ is a *p* × 1 study-specific random effects, and **e**_*i*_ is a *p*_*i*_ × 1 sampling error.

We assume that Var(**e**_*i*_) = **V**_*i*_ is known in the *i*th study and that Var(**u**_*i*_) = **T**^2^ is the variance component of the between-study heterogeneity that has to be estimated. The model handles missing effect sizes by selecting the complete effect sizes only in the above equation. Since **x**_*i*_ is a design matrix, missing value is not allowed in **x**_*i*_. When there are missing values in **x**_*i*_, the whole study will be deleted before the analysis is conducted.

The −2*LL* of the above model is:

(16) $$\begin{array}{l} -2LL_{i}{\left(B,T^{2};y_{i}\right)}_{\text{ML}}=\;p_{i}*\log\left(2\pi \right)+\log|Z_{i}T^{2}Z_{i}^{⊤}+V_{i}|\\ \;+\;{\left(y_{i}-B_{i}x_{i}\right)}^{⊤}{\left(Z_{i}T^{2}Z_{i}^{⊤}+V_{i}\right)}^{-1}\\ \;\left(y_{i}-B_{i}x_{i}\right). \end{array}$$

To fit the multivariate mixed-effects meta-analysis in SEM, we use the following model implied conditional mean vector and covariance matrix (Cheung, 2013b):

(17) $$\begin{array}{l} \mu_{i}(\theta |x_{i})=B_{i}x_{i}\text{ and}\\ \Sigma_{i}(\theta |x_{i})=Z_{i}T^{2}Z_{i}^{⊤}+V_{i}. \end{array}$$

**Figure 4:**
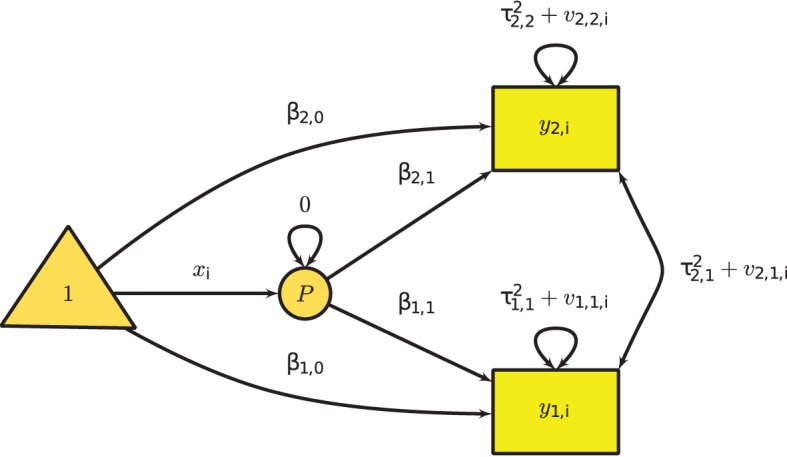
**Multivariate mixed-effects meta-analysis with two effect sizes per study and one predictor**.

Figure 4 shows the graphical model of the multivariate mixed-effects meta-analysis with two effect sizes per study and one predictor. A phantom variable *P* is introduced to specify the predictor **x**_*i*_.

The multivariate random-effects meta-analysis is a special case of the multivariate mixed-effects meta-analysis by using **X**_*i*_ = 1 as the design matrix; the random-effects meta-analysis is a special case of the fixed-effects meta-analysis by fixing **T**^2^ = **0**. Moreover, the univariate meta-analysis is also a special case of the multivariate meta-analysis with only one effect size. The *I*^2^ and *R*^2^ in a univariate meta-analysis may also be calculated for each effect size in a multivariate meta-analysis.

### 2.5. Three-level meta-analysis

Effect sizes are assumed to be independent in most meta-analytic models. However, the effect sizes can be non-independent for various reasons. For example, the effect sizes reported in the same study may be more similar than the effect sizes reported in other studies. When the degree of dependence is known, the multivariate meta-analysis introduced in the Section 2.4 can be used to model the dependence. When the degree of dependence is not known, a three-level meta-analytic model may be used to address the dependence among the effect sizes (e.g., Konstantopoulos, 2011; Cheung, 2014c; Van den Noortgate et al., 2013). The model is:

(18) $$y_{ij}=x_{ij}^{⊤}\beta +u_{(2)ij}+u_{(3)j}+e_{ij},$$

where *y*_*ij*_ is the effect size for the *i*th effect size in the *j*th cluster, β is an (*m* + 1) × 1 vector of regression coefficients including the intercept, **x**_*ij*_ is the (*m* + 1) × 1 predictors including 1 in the first element for the *i*th study at the *j*th cluster, *u*_(2)*ij*_ and *u*_(3)*j*_ are the random effects at level 2 and level 3, respectively, and Var(*e*_*ij*_) = *v*_*ij*_ is the known sampling variance of the effect size.

To fit the three-level meta-analytic model in SEM, we may use the following model implied moments for the conditional mean and variance:

(19) $$\begin{array}{l} \mu_{ij}(\theta |x_{ij})=x_{ij}^{⊤}\beta \text{ and}\\ \Sigma_{ij}(\theta |x_{ij})=\tau_{\left(2\right)}^{2}+\tau_{\left(3\right)}^{2}+v_{ij}, \end{array}$$

where Var(*u*_(2)*ij*_) = τ^2^_(2)_ and Var(*u*_(3)*j*_) = τ^2^_(3)_ are the heterogeneity at level 2 and level 3, respectively (Cheung, 2014c).

#### 2.5.1. Quantifying heterogeneity and explained variance

Similar to the *I*^2^ defined in a random-effects meta-analysis, we may define the degree of heterogeneity for a three-level meta-analysis in level 2 and level 3 as,

(20) $$\begin{array}{l} I_{\left(2\right)}^{2}=\frac{{\hat{\tau }}_{\left(2\right)}^{2}}{{\hat{\tau }}_{\left(2\right)}^{2}+{\hat{\tau }}_{\left(3\right)}^{2}+\tilde{v}}\text{ and}\\ I_{\left(3\right)}^{2}=\frac{{\hat{\tau }}_{\left(3\right)}^{2}}{{\hat{\tau }}_{\left(2\right)}^{2}+{\hat{\tau }}_{\left(3\right)}^{2}+\tilde{v}}, \end{array}$$

where $\tilde{v}$ is the *typical* within-study sampling variance defined in a random-effects meta-analysis. *I*^2^_(2)_ and *I*^2^_(3)_ can be interpreted as the proportion of the total variation of the effect size that is due to the level 2 and level 3, respectively. Since $\tilde{v}$ is sample specific, one limitation of *I*^2^_(2)_ and *I*^2^_(3)_ is that they are not estimating any population quantities. Cheung (2014c) introduced two intra-class correlations (*ICC*s),

(21) $$\begin{array}{l} ICC_{\left(2\right)}=\frac{{\hat{\tau }}_{\left(2\right)}^{2}}{{\hat{\tau }}_{\left(2\right)}^{2}+{\hat{\tau }}_{\left(3\right)}^{2}}\text{ and}\\ ICC_{\left(3\right)}=\frac{{\hat{\tau }}_{\left(3\right)}^{2}}{{\hat{\tau }}_{\left(2\right)}^{2}+{\hat{\tau }}_{\left(3\right)}^{2}}. \end{array}$$

Both *ICC*_(2)_ and *ICC*_(3)_ are estimating their population counterparts τ^2^_(2)_/(τ^2^_(2)_ + τ^2^_(3)_) and τ^2^_(3)_/(τ^2^_(2)_ + τ^2^_(3)_), respectively. *ICC*_(2)_ and *ICC*_(3)_ can be interpreted as the percentage of the population heterogeneity due to level 2 and level 3, respectively.

When there are predictors, we may calculate the *R*^2^ for level 2 and level 3 in a similar manner to that defined before,

(22) $$\begin{array}{l} R_{\left(2\right)}^{2}=\frac{{\hat{\tau }}_{(2)0}^{2}-{\hat{\tau }}_{(2)1}^{2}}{{\hat{\tau }}_{(2)0}^{2}}\text{ and}\\ R_{\left(3\right)}^{2}=\frac{{\hat{\tau }}_{(3)0}^{2}-{\hat{\tau }}_{(3)1}^{2}}{{\hat{\tau }}_{(3)0}^{2}}. \end{array}$$

When the estimates are negative, they are usually truncated to zero.

## 3. Meta-analytic structural equation modeling

SEM is a popular modeling techniques in the social and behavioral sciences. When there are more and more studies addressing similar research questions using similar variables, there is a need to compare and synthesize these findings. MASEM combines ideas of meta-analysis and SEM by pooling correlation (or covariance) matrices and testing structural equation models on the pooled correlation (or covariance) matrix (e.g., Viswesvaran and Ones, 1995; Cheung and Chan, 2005; Becker, 2009). There are two stages in conducting the analysis. In the first stage of the analysis, the correlation (or covariance) matrices are pooled together. In the second stage of the analysis, the pooled correlation (or covariance) matrix is used to fit structural equation models.

Cheung and Chan (2005, 2009) proposed a fixed-effects TSSEM. The fixed-effects TSSEM approach has been extended to the random-effects TSSEM by Cheung (2014a). Regardless of whether a fixed- or a random-effects model is used, the metaSEM package handles this automatically. In other words, parameter estimates, *SE*s, and goodness-of-fit indices in the stage 2 analysis have already taken the stage 1 model into account.

### 3.1. Stage 1 analysis

The main objective of the stage 1 analysis is to pool the correlation (or covariance) matrices together. There are two classes of models in meta-analysis—fixed-effects models and random-effects models (see Hedges and Vevea, 1998; Schmidt et al., 2009). Fixed-effects models are used for conditional inferences based on the selected studies. They are intended to draw conclusions on the studies included in the meta-analysis. Researchers are mainly interested in the studies used in the analysis. The assumption in fixed-effects models is usually, but not always, that all studies share common effect sizes. The stage one analysis in both the fixed- and the random-effects TSSEM is based on the ML estimation method. Thus, the parameter estimates are unbiased and efficient when the missing correlation coefficients are missing completely at random (MCAR) or missing at random (MAR) (e.g., Enders, 2010).

#### 3.1.1. Fixed-effects model

Under the fixed-effects (or more correctly the common effects) model, it is assumed that the population correlation (or covariance) matrices are the same while there are study-specific correlation (or covariance matrices) under the random-effects model. To simplify the presentation, I will mainly focus on the analysis of correlation matrices. Generalizing to analysis of covariance matrices is a straight-forward process (see Cheung and Chan, 2009). A covariance matrix in the *i*th study can be decomposed into a product of the matrices of correlations and standard deviations:

(23) $$\Sigma_{i}(\theta )=D_{i}P_{i}D_{i},$$

where Σ_*i*_(θ) is the model implied covariance matrix, **D**_*i*_ is the diagonal matrix of standard deviations, and **P**_*i*_ is the correlation matrix. Under the assumption of the homogeneity of correlation matrices, we may obtain a common correlation matrix by imposing the constraint *P* = *P*_1_ = *P*_2_ = … = *P*_*k*_, where *D*_*i*_ may vary across studies. When there are missing correlations, the missing data are filtered out. If we want to obtain a common covariance matrix under the assumption of the homogeneity of covariance matrices, we may also add the constraint *D* = *D*_1_ = *D*_2_ = … = *D*_*k*_.

An *LR* statistic can be used to test the null hypothesis of homogeneity of correlation matrices *P*_1_ = *P*_2_ = … = *P*_*k*_. Moreover, various goodness-of-fit indices may also be used to evaluate the appropriateness of the “close” fit of the homogeneity of correlation matrices.

#### 3.1.2. Random-effects model

Since the primary studies are independently conducted by different researchers, the samples, measures, and research focuses are likely different. The assumption of homogeneity of correlation matrices may not be reasonable. A random-effects TSSEM is usually more appropriate to analyze the data (Cheung, 2014a). When a random-effects model is used, the correlation matrices are treated as vectors of multivariate effect sizes. Let **r**_*i*_ = vechs(**R**_*i*_) be the *p*(*p* − 1)/2 × 1 vector of a sample correlation for *p* variables by taking the column-wise non-redundant elements from **R**_*i*_. If an analysis of the covariance matrices is conducted, the *p*(*p* + 1)/2 × 1 vectorized multivariate effect sizes become **s**_*i*_ = vech(**S**_*i*_).

The model for the sample correlation vector **r**_*i*_ is:

(24) $$r_{i}=\rho_{\text{R}}+u_{i}+e_{i},$$

where ρ_R_ is the *p*(*p* − 1)/2 × 1 vector of average population correlation vector under a random-effects model, Var(**u**_*i*_) = **T**^2^ is the variance components of the random effects, and Var(**e**_*i*_) = **V**_*i*_ is the known conditional sampling covariance matrix. The multivariate random-effects meta-analysis introduced in Section 2.4 may be used to conduct the stage 1 analysis with a random-effects model.

When there are many variables or not enough data (studies) in the analysis, $\hat{T}$^2^ can be non-positive definite. The results cannot be trusted. One workaround is to fix **T**^2^ to a diagonal matrix rather than as a symmetric matrix. This can be done easily by specifying the argument RE.type = “Diag” when calling the tssem1() function.

### 3.2. Stage 2 analysis

After the stage 1 analysis with either a fixed- or a random-effects model, a vector of pooled correlations **r** and its asymptotic covariance matrix **V** are available after the analysis. It should be noted that $\hat{T}$^2^ is not directly involved in fitting the correlation structure in the stage 2 analysis. However, the presence of **T**^2^ is required so that the heterogeneity of the random effects has been properly taken in the stage 1 analysis.

Most applications of MASEM use the pooled correlation matrix as if it was an observed correlation matrix to fit structural equation models. Cheung and Chan (2005) discussed some of these problems. For example, the elements of the pooled correlation matrix are usually based on different studies. Researchers usually use an *ad-hoc* sample size, such as the harmonic or arithmetic means of the individual sample sizes, as the sample size in fitting structural equation models. Unless all the correlation coefficients are based on the same number of studies, the precision of some correlation coefficients are over-estimated while others are under-estimated. Another issue is that the pooled correlation matrix is analyzed as it was a covariance matrix. It is generally incorrect to analyze the correlation matrix in SEM, although most published articles using MASEM have treated the pooled correlation matrix as a covariance matrix. Many SEM experts (e.g., Cudeck, 1989) have warned about the problems of analyzing the correlation matrix instead of the covariance matrix in primary-research applications of SEM. Specifically, the chi-square statistics and (or) the *SE*s of parameter estimates may be incorrect.

The TSSEM approach addresses these issues. The weighted least square (WLS) estimation is used to fit the proposed models in the stage 2 analysis. A correlation structural model ρ($\hat{\gamma }$) is fitted with the WLS estimation method by minimizing the following fit function,

(25) $$F(\hat{\gamma })={\left(r-\rho (\hat{\gamma })\right)}^{⊤}V^{-1}(r-\rho (\hat{\gamma })).$$

An *LR* statistic and various goodness-of-fit indices may be used to judge whether the proposed structural model is appropriate, while *SE*s may be used to test the significance of individual parameter estimates.

## 4. Conclusion

This paper introduced various meta-analytic models using a SEM approach. More importantly, these models have all been implemented in the metaSEM package which is freely available as an R package. Due to space constraint, I did not include topics, such as using REML as the estimation method (see Cheung, 2013a), constructing likelihood-based confidence interval (LBCI) (see Cheung, 2009), and using alternative random-effects MASEM (see Cheung and Cheung, under review). Readers may refer to the relevant papers and the metaSEM package for details. Some of these models can be implemented in standard SEM software such as M*plus* (Muthén and Muthén, 2012). Since SEM software was not designed for meta-analysis, transformations on the effect sizes are required to meet the distribution assumptions (see e.g., Cheung, 2008, 2013b). To conclude, SEM provides a flexible framework to develop meta-analytic techniques. Many of the techniques available in SEM can be easily extended to meta-analysis. The supplementary material include some examples on how to analyze these models using the metaSEM package.

## Funding

Preparation of this paper was supported by the Academic Research Fund Tier 1 (FY2013-FRC5-002; R-581-000-158-112) from the Ministry of Education, Singapore.

### Conflict of interest statement

The author declares that the research was conducted in the absence of any commercial or financial relationships that could be construed as a potential conflict of interest.
