# Supplementary material for: metaSEM: an R package for meta-analysis using structural equation modeling
Source: Front Psychol. 2015 Jan 5;5:1521. doi: 10.3389/fpsyg.2014.01521 (PMC4283449; doi:10.3389/fpsyg.2014.01521)
Supplement: Supplementary file 1 [file Presentation1.PDF]

Frontiers in Psychology

Method Article
26 November 2014

Supplementary Material: metaSEM: An R Package for Meta-Analysis using Structural Equation Modeling

Mike W.-L. Cheung [1,*]

[1] *Department of Psychology, National University of Singapore, Singapore 117570*

Correspondence*:
Mike W.-L. Cheung
Department of Psychology, National University of Singapore, Singapore 117570,
mikewlcheung@nus.edu.sg

1 INTRODUCTION

Several examples are used to demonstrate the procedures of fitting various meta-analyses and meta-analytic structural equation modeling (MASEM) using the metaSEM package implemented in R. All the data sets are stored in the metaSEM package. We may access the data by calling library("metaSEM") in R.

2 UNIVARIATE META-ANALYSIS

2.1 EXAMPLE 1

Becker (1983) reported 10 studies on sex differences in conformity using the fictitious norm group paradigm. di and vi are the standardized mean difference and its sampling variance, respectively. percentage and items are the percentage of male authors and the number of items, respectively. We need to load the metaSEM package before calling the functions in the package.

```
## Load the library
library("metaSEM")

## Display the content of the data
Becker83

##    study    di   vi percentage items
## 1      1 -0.33 0.03         25     2
## 2      2  0.07 0.03         25     2
## 3      3 -0.30 0.02         50     2
## 4      4  0.35 0.02        100    38
## 5      5  0.69 0.07        100    30
## 6      6  0.81 0.22        100    45
## 7      7  0.40 0.05        100    45
## 8      8  0.47 0.07        100    45
## 9      9  0.37 0.05        100     5
```

```
## 10    10 -0.06 0.03         100      5
```

2.1.1 Univariate random-effects model The function `meta()` is used to conduct the univariate and multivariate meta-analyses. The arguments `y` and `v` are used to specify the effect sizes and their sampling variances, respectively. By default, a random-effects meta-analysis is fitted. After running the analysis, `summary()` is used to extract the results.

```
summary( meta(y=di, v=vi, data=Becker83) )

##
## Call:
## meta(y = di, v = vi, data = Becker83)
##
## 95% confidence intervals: z statistic approximation
## Coefficients:
##              Estimate Std.Error  lbound  ubound z value Pr(>|z|)
## Intercept1   0.1747    0.1134 -0.0475  0.3969    1.54     0.12
## Tau2_1_1     0.0774    0.0541 -0.0287  0.1834    1.43     0.15
##
## Q statistic on the homogeneity of effect sizes: 30.65
## Degrees of freedom of the Q statistic: 9
## P value of the Q statistic: 0.0003399
##
## Heterogeneity indices (based on the estimated Tau2):
##                              Estimate
## Intercept1: I2 (Q statistic)     0.67
##
## Number of studies (or clusters): 10
## Number of observed statistics: 10
## Number of estimated parameters: 2
## Degrees of freedom: 8
## -2 log likelihood: 7.928
## OpenMx status1: 0 ("0" or "1": The optimization is considered fine.
## Other values indicate problems.)
```

Before interpreting the results, we must check whether the optimization was successful. The `OpenMx status1` returns the status from the optimizer. The optimization can be considered to be fine if the code is either 0 or 1. Users may refer to OpenMx's Common Errors (and how to avoid them) for more details. From the output, the Q statistic $(df = 9)$ is 30.65, $p < 0.001$. The estimated heterogeneity variance is 0.0774, while the I^2 based on the Q statistic is 0.67. The average effect size with its 95% Wald confidence interval (CI) based on the random-effects model is 0.1747 (-0.0475, 0.3969).

2.1.2 Univariate mixed-effects model Following (**Becker**, 1983), we may conduct a mixed-effects meta-analysis by including `log(items)` as a moderator. The argument `x` is used to specify the predictors. The estimated regression coefficients are represented by the `Slopei_j` parameter, where i and j represent the ith effect size and the jth predictor in the output.

```
summary( meta(y=di, v=vi, x=log(items), data=Becker83) )

##
## Call:
## meta(y = di, v = vi, x = log(items), data = Becker83)
##
## 95% confidence intervals: z statistic approximation
## Coefficients:
##              Estimate Std.Error    lbound    ubound z value Pr(>|z|)
## Intercept1 -3.20e-01  1.10e-01 -5.35e-01 -1.05e-01   -2.92   0.0036
## Slope1_1    2.11e-01  4.51e-02  1.23e-01  2.99e-01    4.68  2.9e-06
## Tau2_1_1    1.00e-10  2.01e-02 -3.94e-02  3.94e-02    0.00   1.0000
##
## Intercept1 **
## Slope1_1   ***
## Tau2_1_1
## ---
## Signif. codes:  0 '***' 0.001 '**' 0.01 '*' 0.05 '.' 0.1 ' ' 1
##
## Q statistic on the homogeneity of effect sizes: 30.65
## Degrees of freedom of the Q statistic: 9
## P value of the Q statistic: 0.0003399
##
## Explained variances (R2):
##                           y1
## Tau2 (no predictor)     0.08
## Tau2 (with predictors) 0.00
## R2                      1.00
##
## Number of studies (or clusters): 10
## Number of observed statistics: 10
## Number of estimated parameters: 3
## Degrees of freedom: 7
## -2 log likelihood: -4.208
## OpenMx status1: 0 ("0" or "1": The optimization is considered fine.
## Other values indicate problems.)
```

The result shows that `log(items)` is a significant predictor with the estimated regression coefficient and its 95% Wald CI of 0.211 (0.123, 0.299) with $R^2 = 1$. This suggests that the effect sizes in the studies become larger when there are more items used to measure the constructs.

2.1.3 Univariate fixed-effects model Mathematically, the fixed-effects meta-analysis is a special case of the random-effects meta-analysis by fixing the heterogeneity variance of the random-effects at 0. The argument `RE.constraints` is used to constrain the variance component of the random effects. The following analysis shows that the estimated common effect and its 95% Wald CI under a fixed-effects model is 0.1006 (-0.0180, 0.2192).

```
summary( meta(y=di, v=vi, data=Becker83, RE.constraints=0) )

##
```

```
## Call:
## meta(y = di, v = vi, data = Becker83, RE.constraints = 0)
##
## 95% confidence intervals: z statistic approximation
## Coefficients:
##              Estimate Std.Error  lbound  ubound z value Pr(>|z|)
## Intercept1    0.1006    0.0605 -0.0180  0.2192    1.66    0.096 .
## ---
## Signif. codes:  0 '***' 0.001 '**' 0.01 '*' 0.05 '.' 0.1 ' ' 1
##
## Q statistic on the homogeneity of effect sizes: 30.65
## Degrees of freedom of the Q statistic: 9
## P value of the Q statistic: 0.0003399
##
## Heterogeneity indices (based on the estimated Tau2):
##                               Estimate
## Intercept1: I2 (Q statistic)        0
##
## Number of studies (or clusters): 10
## Number of observed statistics: 10
## Number of estimated parameters: 1
## Degrees of freedom: 9
## -2 log likelihood: 17.86
## OpenMx status1: 0 ("0" or "1": The optimization is considered fine.
## Other values indicate problems.)
```

2.2 EXAMPLE 2

Jaramillo et al. (2005) conducted a meta-analysis of 61 studies on the relationship between organizational commitment and salesperson job performance. The effect size was a correlation coefficient. **Jaramillo et al.** (2005) corrected for unreliability before conducting the analysis. As an illustration, we use the uncorrected correlation coefficients here. The effect size and its sampling variance are `r` and `r_v`, respectively.

```
## Show the first few cases
head(Jaramillo05)

##                          Author Sample_size    Sales Country IDV
## 1          Aryee et al. (2002)         179    mixed   India  48
## 2 Balfour and Wechsler (1991)         232 nonsales     USA  91
## 3     Bashaw and Grant (1994)         560    sales     USA  91
## 4              Benkhoff (1997)         181    sales Germany  67
## 5          Brett et al. (1995)         156    sales     USA  91
## 6          Brett et al. (1995)         180    sales     USA  91
##          OC_scale OC_alpha JP_alpha    r      r_v
## 1 Porter or Mowday     0.87     0.89 0.02 0.005582
## 2            other     0.82       NA 0.12 0.004187
## 3 Porter or Mowday     0.83     0.76 0.09 0.001757
## 4 Porter or Mowday       NA     1.00 0.20 0.005092
## 5 Porter or Mowday     0.83       NA 0.08 0.006328
```

```
## 6 Porter or Mowday      0.83       NA 0.04 0.005538
```

2.2.1 Random-effects model We employ a random-effects model with the following syntax. By default, the I^2 is calculated based on the Q statistic (with the `I2="I2q"` argument in calling the `meta()` function). Readers can also use either the harmonic mean (`I2="I2hm"`) or the arithmetic mean (`I2="I2am"`) of the sampling variances to calculate the I^2.

```
summary( meta(y=r, v=r_v, data=Jaramillo05) )

##
## Call:
## meta(y = r, v = r_v, data = Jaramillo05)
##
## 95% confidence intervals: z statistic approximation
## Coefficients:
##             Estimate Std.Error  lbound  ubound z value Pr(>|z|)
## Intercept1  0.18662   0.01933 0.14874 0.22451    9.65  < 2e-16 ***
## Tau2_1_1    0.01703   0.00414 0.00893 0.02514    4.12  3.8e-05 ***
## ---
## Signif. codes:  0 '***' 0.001 '**' 0.01 '*' 0.05 '.' 0.1 ' ' 1
##
## Q statistic on the homogeneity of effect sizes: 339.4
## Degrees of freedom of the Q statistic: 60
## P value of the Q statistic: 0
##
## Heterogeneity indices (based on the estimated Tau2):
##                              Estimate
## Intercept1: I2 (Q statistic)     0.81
##
## Number of studies (or clusters): 61
## Number of observed statistics: 61
## Number of estimated parameters: 2
## Degrees of freedom: 59
## -2 log likelihood: -55.44
## OpenMx status1: 0 ("0" or "1": The optimization is considered fine.
## Other values indicate problems.)
```

The homogeneity test of effect sizes is statistically significant with $Q(df = 60) = 339.4, p < 0.001$. The $\hat{\tau}^2 = .0170$ and $I^2 = 0.81$. These indicate that there is a high degree of heterogeneity. The between-study effect explains 81% of the total variation. The estimated average population correlation coefficient (with its 95% Wald CI) based on a random-effects model is 0.1866 (0.1487, 0.2245).

2.2.2 Likelihood-based CI The above CIs are based on the Wald approximation (labelled as a `z statistic approximation` in the output). When the number of studies is small, LBCI (labelled as a `Likelihood-based statistic` in the output) is preferred (e.g., **Cheung**, 2009; **Neale and Miller**, 1997). We may request the LBCI by specifying the `intervals.type="LB"` argument. Since I^2 is a function of $\hat{\tau}^2$, LBCI on I^2 is also reported. The 95% LBCIs on $\hat{\tau}^2$ and I^2 are (0.0106, 0.0276), and (0.732, 0.880), respectively.

```
summary( meta(y=r, v=r_v, data=Jaramillo05, intervals.type="LB") )

##
## Call:
## meta(y = r, v = r_v, data = Jaramillo05, intervals.type = "LB")
##
## 95% confidence intervals: Likelihood-based statistic
## Coefficients:
##            Estimate Std.Error lbound ubound z value Pr(>|z|)
## Intercept1   0.1866        NA 0.1480 0.2251      NA       NA
## Tau2_1_1     0.0170        NA 0.0106 0.0276      NA       NA
##
## Q statistic on the homogeneity of effect sizes: 339.4
## Degrees of freedom of the Q statistic: 60
## P value of the Q statistic: 0
##
## Heterogeneity indices (I2) and their 95% likelihood-based CIs:
##                              lbound Estimate ubound
## Intercept1: I2 (Q statistic)  0.732    0.814   0.88
##
## Number of studies (or clusters): 61
## Number of observed statistics: 61
## Number of estimated parameters: 2
## Degrees of freedom: 59
## -2 log likelihood: -55.44
## OpenMx status1: 0 ("0" or "1": The optimization is considered fine.
## Other values indicate problems.)
```

2.2.3 Mixed-effects model The moderators can be included by specifying the `x` argument in the `meta()` function. When there is more than one moderators, they can be combined using the `cbind()` command. The explained variance R^2 on the effect size is also reported.

The dataset includes the coefficient alpha of the scales on measuring organizational commitment and job performance (`OC_alpha` and `JP_alpha` in the dataset). As an illustration, we include both `OC_alpha` and `JP_alpha` as the moderators.

```
## Label this model as "Unequal coefficients"
model1 <- meta(y=r, v=r_v, x=cbind(OC_alpha, JP_alpha),
               data=Jaramillo05,
               model.name="Unequal coefficients")
summary(model1)

##
## Call:
## meta(y = r, v = r_v, x = cbind(OC_alpha, JP_alpha), data = Jaramillo05,
##     model.name = "Unequal coefficients")
##
## 95% confidence intervals: z statistic approximation
## Coefficients:
##            Estimate Std.Error   lbound   ubound z value Pr(>|z|)
```

```
## Intercept1 -0.57554   0.50152 -1.55849  0.40742   -1.15  0.25114
## Slope1_1    0.13110   0.45872 -0.76797  1.03018    0.29  0.77503
## Slope1_2    0.80442   0.43038 -0.03912  1.64796    1.87  0.06161 .
## Tau2_1_1    0.01873   0.00565  0.00765  0.02981    3.31  0.00092 ***
## ---
## Signif. codes:  0 '***' 0.001 '**' 0.01 '*' 0.05 '.' 0.1 ' ' 1
##
## Q statistic on the homogeneity of effect sizes: 257.7
## Degrees of freedom of the Q statistic: 34
## P value of the Q statistic: 0
##
## Explained variances (R2):
##                           y1
## Tau2 (no predictor)     0.02
## Tau2 (with predictors) 0.02
## R2                      0.00
##
## Number of studies (or clusters): 35
## Number of observed statistics: 35
## Number of estimated parameters: 4
## Degrees of freedom: 31
## -2 log likelihood: -31.01
## OpenMx status1: 0 ("0" or "1": The optimization is considered fine.
## Other values indicate problems.)
```

The estimated regression coefficients for `OC_alpha` and `JP_alpha` (`Slope1_1` and `Slope1_2` in the output) are $\hat{\beta}_{\text{OC}_{\text{alpha}}} = 0.1311$, $SE_{\text{OC}_{\text{alpha}}} = 0.4587$, $p_{\text{OC}_{\text{alpha}}} = 0.7750$, and $\hat{\beta}_{\text{JP}_{\text{alpha}}} = 0.8044$, $SE_{\text{JP}_{\text{alpha}}} = 0.4304$, $p_{\text{JP}_{\text{alpha}}} = 0.0616$, respectively. Neither of them is statistically significant at $\alpha = 0.05$ and the $R^2 = 0$. Therefore, there is no evidence indicating that the reliabilities of the measures are correlated with the effect size.

Although both coefficients are non-significant in the above analysis, we test $H_0 : \beta_{\text{equal}} = \beta_{\text{OC}_{\text{alpha}}} = \beta_{\text{JP}_{\text{alpha}}}$ as an illustration. First, we need to fit a model with an equality constraint on the regression coefficients by specifying the `coef.constraints` argument. The argument expects a $p \times m$ matrix, where p is the number of effect sizes and m is the number of predictors. In this example, it is 1×2 matrix, where the first and second elements refer to the regression coefficients of `OC_alpha`, and `JP_alpha`, respectively.

We may impose the equality constraint by using the same label in the constraint. In this example, `0*` represents the starting value for the regression coefficients while `Slope_equal` is the name of both coefficients. We further call this model `model.name="Equal slopes"` for ease of comparison and save the results to an R object called `model2`.

```
( constraint <- matrix(c("0*Slope_equal", "0*Slope_equal"),
                        nrow=1, ncol=2) )

##      [,1]            [,2]
## [1,] "0*Slope_equal" "0*Slope_equal"
```

```
model2 <- meta(y=r, v=r_v, x=cbind(OC_alpha, JP_alpha),
               data=Jaramillo05, coef.constraints=constraint,
               model.name="Equal coefficients")
summary(model2)

##
## Call:
## meta(y = r, v = r_v, x = cbind(OC_alpha, JP_alpha), data = Jaramillo05,
##     coef.constraints = constraint, model.name = "Equal coefficients")
##
## 95% confidence intervals: z statistic approximation
## Coefficients:
##              Estimate Std.Error   lbound   ubound z value Pr(>|z|)
## Intercept1   -0.60367   0.50745 -1.59826  0.39092   -1.19  0.23420
## Slope_equal   0.48630   0.29531 -0.09249  1.06509    1.65  0.09961 .
## Tau2_1_1      0.01937   0.00582  0.00796  0.03079    3.33  0.00088 ***
## ---
## Signif. codes:  0 '***' 0.001 '**' 0.01 '*' 0.05 '.' 0.1 ' ' 1
##
## Q statistic on the homogeneity of effect sizes: 257.7
## Degrees of freedom of the Q statistic: 34
## P value of the Q statistic: 0
##
## Explained variances (R2):
##                            y1
## Tau2 (no predictor)      0.02
## Tau2 (with predictors) 0.02
## R2                       0.00
##
## Number of studies (or clusters): 35
## Number of observed statistics: 35
## Number of estimated parameters: 3
## Degrees of freedom: 32
## -2 log likelihood: -30.02
## OpenMx status1: 0 ("0" or "1": The optimization is considered fine.
## Other values indicate problems.)
```

The estimated constrained regression coefficient is $\hat{\beta}_{\text{equal}} = 0.4863, SE_{\text{equal}} = 0.2953, p_{\text{equal}} = 0.10$, which is still non-significant. To test $H_0 : \beta_{\text{OC}_{\text{alpha}}} = \beta_{\text{JP}_{\text{alpha}}}$, we compare `model1` against `model2` with the `anova()` function.

```
anova(model1, model2)

##                    base         comparison ep minus2LL df    AIC
## 1 Unequal coefficients               <NA>  4   -31.01 31 -93.01
## 2 Unequal coefficients Equal coefficients  3   -30.02 32 -94.02
##   diffLL diffdf      p
## 1     NA     NA     NA
## 2 0.9943      1 0.3187
```

The *LR* statistic is $\Delta\chi^2(df = 1) = 0.9943, p = 0.3187$. Therefore, there is not enough evidence to reject the null hypothesis of equal regression coefficients.

2.2.4 Testing categorical predictors There are three types of samples in the data set: `sales`, `nonsales`, and `mixed` in the variable `Sales`. A typical approach is to use one group, say `nonsales`, as the reference group and create two dummy variables ($\mathbf{D}_{\text{sales}}$ and $\mathbf{D}_{\text{mixed}}$) with only 0 and 1 for `sales` and `mixed` to represent the differences between these groups to the reference group, the `nonsales`. The model is:

$$\mathbf{y} = \beta_0 + \beta_1\mathbf{D}_{\text{sales}} + \beta_2\mathbf{D}_{\text{mixed}} + \mathbf{u} + \mathbf{e}, \quad (1)$$

where β_0 is the population effect size for `nonsales`, β_1 is the difference between `sales` and `nonsales`, and β_2 is the difference between `mixed` and `nonsales`.

Although the model can be used to test the differences among the groups, it does not provide the estimates for all groups. An alternative approach is to create three indicator variables. We may fit a model without an intercept:

$$\mathbf{y} = \beta_1\mathbf{D}_{\text{sales}} + \beta_2\mathbf{D}_{\text{mixed}} + \beta_3\mathbf{D}_{\text{nonsales}} + \mathbf{u} + \mathbf{e}, \quad (2)$$

where β_1, β_2, and β_3 now represent the average population effect sizes for `sales`, `mixed`, and `nonsales`, respectively. In order to estimate the means for all three groups, the intercept must be fixed at 0; otherwise, the model is not identified. To test whether all group means are the same, we compare the above model against the intercept model. Under the null hypothesis $H_0 : \beta_1 = \beta_2 = \beta_3$, the test statistic has a chi-square distribution with $df = 2$.

First, we show the frequency table of the variable `Sales`. Then, we create three indicator variables by using the `ifelse()` command.

```
table(Jaramillo05$Sales)

##
##    mixed nonsales    sales
##        6       27       28

sales <- ifelse(Jaramillo05$Sales=="sales", yes=1, no=0)
nonsales <- ifelse(Jaramillo05$Sales=="nonsales", yes=1, no=0)
mixed <- ifelse(Jaramillo05$Sales=="mixed", yes=1, no=0)
```

To fit the model without an intercept, we fix the intercept at 0 by specifying the `intercept.constraints=0` argument. Since the original starting values assume that there is an intercept, there were estimation problems in the model without the intercept. We provide starting values for the regression coefficients by using the `coef.constraints` argument:

```
( startvalues <- matrix(c("0*Slope1_1", "0*Slope1_2",
                          "0*Slope1_3"), nrow=1, ncol=3) )

##      [,1]         [,2]         [,3]
## [1,] "0*Slope1_1" "0*Slope1_2" "0*Slope1_3"
```

```
model3 <- meta(y=r, v=r_v, x=cbind(sales, mixed, nonsales),
               data=Jaramillo05, coef.constraints=startvalues,
               intercept.constraints=0,
```

```
                  model.name="Indicator variables")
summary(model3)

##
## Call:
## meta(y = r, v = r_v, x = cbind(sales, mixed, nonsales), data = Jaramillo0
##     intercept.constraints = 0, coef.constraints = startvalues,
##     model.name = "Indicator variables")
##
## 95% confidence intervals: z statistic approximation
## Coefficients:
##            Estimate Std.Error  lbound  ubound z value Pr(>|z|)
## Slope1_1  0.22830   0.02759 0.17421 0.28238    8.27  2.2e-16 ***
## Slope1_2  0.14659   0.06328 0.02257 0.27061    2.32    0.021 *
## Slope1_3  0.15196   0.02794 0.09720 0.20672    5.44  5.4e-08 ***
## Tau2_1_1  0.01573   0.00385 0.00818 0.02328    4.08  4.4e-05 ***
## ---
## Signif. codes:  0 '***' 0.001 '**' 0.01 '*' 0.05 '.' 0.1 ' ' 1
##
## Q statistic on the homogeneity of effect sizes: 339.4
## Degrees of freedom of the Q statistic: 60
## P value of the Q statistic: 0
##
## Explained variances (R2):
##                           y1
## Tau2 (no predictor)     0.02
## Tau2 (with predictors) 0.02
## R2                      0.08
##
## Number of studies (or clusters): 61
## Number of observed statistics: 61
## Number of estimated parameters: 4
## Degrees of freedom: 57
## -2 log likelihood: -59.56
## OpenMx status1: 0 ("0" or "1": The optimization is considered fine.
## Other values indicate problems.)
```

The estimated average effects and their 95% Wald CIs for the `sales`, `mixed`, and `nonsales` are 0.2283 (0.1742, 0.2824), 0.1466 (0.0226, 0.2706), and 0.1520 (0.0972, 0.2067), respectively. All of them are statistically significant at $\alpha = .05$.

When the null hypothesis $H_0 : \beta_1 = \beta_2 = \beta_3$ is true, this model is equivalent to the model with only an intercept. Since the model with only an intercept `model4` is nested within the model with predictors `model3`, we compare them with the following code:.

```
model4 <- meta(y=r, v=r_v, data=Jaramillo05,
               model.name="Null hypothesis")
anova(model3, model4)

##                    base      comparison ep minus2LL df    AIC diffLL
## 1 Indicator variables            <NA>  4   -59.56 57 -173.6     NA
```

```
## 2 Indicator variables Null hypothesis  2   -55.44 59 -173.4  4.114
##   diffdf      p
## 1     NA     NA
## 2      2 0.1278
```

The *LR* statistic is $\Delta\chi^2(df = 2) = 4.1140, p = 0.1278$. Therefore, there is not enough evidence to reject the null hypothesis of equal population correlations. When there are missing values in the moderators, effect sizes with the missing values are deleted before conducting the analyses. The numbers of studies may be different in model comparisons. Users must make sure that the same studies are used in the model comparisons.

3 MULTIVARIATE META-ANALYSIS

3.1 EXAMPLE 1

This data set was adapted from **Berkey et al.** (1998) that compared surgical and non-surgical treatments for medium-severity periodontal disease one year after treatment. The effect sizes are `PD`, and `AL`, while their sampling variance-covariance matrix is `var_PD`, `cov_PD_AL`, and `var_AL`. A multivariate meta-analysis can be fitted by specifying the multivariate effect sizes and their sampling covariance matrix in the arguments `y` and `v` with `cbind()`, respectively. Only the lower triangle of the sampling covariance matrix arranged by the column major is used in `v`. For example, if there are three effect sizes and $\mathbf{V}_i = \begin{bmatrix} V_{11} & & \\ V_{21} & V_{22} & \\ V_{31} & V_{32} & V_{33} \end{bmatrix}$, we may use `meta(y=cbind(y1,y2,y3), v=cbind(V11,V21,V31,V22,V32,V33))`. The following syntax conducts a multivariate random-effects meta-analysis on `Berkey98`:

```
## Display the content of the data
Berkey98

##   trial pub_year no_of_patients   PD    AL var_PD cov_PD_AL var_AL
## 1     1     1983             14 0.47 -0.32 0.0075    0.0030 0.0077
## 2     2     1982             15 0.20 -0.60 0.0057    0.0009 0.0008
## 3     3     1979             78 0.40 -0.12 0.0021    0.0007 0.0014
## 4     4     1987             89 0.26 -0.31 0.0029    0.0009 0.0015
## 5     5     1988             16 0.56 -0.39 0.0148    0.0072 0.0304

summary( meta(y=cbind(PD,AL), v=cbind(var_PD,cov_PD_AL,var_AL),
              data=Berkey98, model.name="Random effects model") )

##
## Call:
## meta(y = cbind(PD, AL), v = cbind(var_PD, cov_PD_AL, var_AL),
##     data = Berkey98, model.name = "Random effects model")
##
## 95% confidence intervals: z statistic approximation
```

```
## Coefficients:
##               Estimate Std.Error   lbound   ubound z value Pr(>|z|)
## Intercept1  0.34484   0.05363  0.23972  0.44995    6.43  1.3e-10 ***
## Intercept2 -0.33794   0.08125 -0.49718 -0.17870   -4.16  3.2e-05 ***
## Tau2_1_1    0.00700   0.00905 -0.01074  0.02474    0.77     0.44
## Tau2_2_1    0.00946   0.00997 -0.01008  0.02900    0.95     0.34
## Tau2_2_2    0.02614   0.01774 -0.00863  0.06092    1.47     0.14
## ---
## Signif. codes:  0 '***' 0.001 '**' 0.01 '*' 0.05 '.' 0.1 ' ' 1
##
## Q statistic on the homogeneity of effect sizes: 128.2
## Degrees of freedom of the Q statistic: 8
## P value of the Q statistic: 0
##
## Heterogeneity indices (based on the estimated Tau2):
##                               Estimate
## Intercept1: I2 (Q statistic)     0.60
## Intercept2: I2 (Q statistic)     0.92
##
## Number of studies (or clusters): 5
## Number of observed statistics: 10
## Number of estimated parameters: 5
## Degrees of freedom: 5
## -2 log likelihood: -11.68
## OpenMx status1: 0 ("0" or "1": The optimization is considered fine.
## Other values indicate problems.)
```

3.1.1 Multivariate random-effects model The Q statistic $(df = 8)$ of the above example is 128.2, $p < 0.001$. The estimated variance component is $\begin{bmatrix} 0.0070 & \\ 0.0095 & 0.02614 \end{bmatrix}$. The I^2 based on the Q statistic for PD and AL are .6021 and .9250, respectively. The pooled effect sizes with their 95% Wald CIs based on the random-effects model for PD and AL are 0.3448 (0.2397, 0.4500), and -0.3379 (-0.4972, -0.1787), respectively.

3.1.2 Multivariate mixed-effects model As an illustration, we use pub_year as a predictor. To make the intercept more interpretable, we center the publication year at 1979, the first year of publication recorded in the data set.

```
mult2 <- meta(y=cbind(PD,AL), v=cbind(var_PD,cov_PD_AL,var_AL),
              data=Berkey98, x=scale(pub_year,center=1979),
              model.name="No constraint")
summary(mult2)

##
## Call:
## meta(y = cbind(PD, AL), v = cbind(var_PD, cov_PD_AL, var_AL),
##     x = scale(pub_year, center = 1979), data = Berkey98, model.name = "No
##
## 95% confidence intervals: z statistic approximation
## Coefficients:
```

```
##              Estimate Std.Error   lbound   ubound z value Pr(>|z|)
## Intercept1  0.34400   0.08577  0.17590  0.51210    4.01    6e-05 ***
## Intercept2 -0.29182   0.13128 -0.54912 -0.03451   -2.22    0.026 *
## Slope1_1    0.00635   0.10782 -0.20498  0.21768    0.06    0.953
## Slope2_1   -0.07059   0.16210 -0.38829  0.24711   -0.44    0.663
## Tau2_1_1    0.00804   0.01012 -0.01180  0.02788    0.79    0.427
## Tau2_2_1    0.00934   0.01055 -0.01134  0.03002    0.89    0.376
## Tau2_2_2    0.02501   0.01708 -0.00846  0.05849    1.46    0.143
## ---
## Signif. codes:  0 '***' 0.001 '**' 0.01 '*' 0.05 '.' 0.1 ' ' 1
##
## Q statistic on the homogeneity of effect sizes: 128.2
## Degrees of freedom of the Q statistic: 8
## P value of the Q statistic: 0
##
## Explained variances (R2):
##                              y1   y2
## Tau2 (no predictor)     0.00700 0.03
## Tau2 (with predictors) 0.00804 0.03
## R2                      0.00000 0.04
##
## Number of studies (or clusters): 5
## Number of observed statistics: 10
## Number of estimated parameters: 7
## Degrees of freedom: 3
## -2 log likelihood: -12.01
## OpenMx status1: 0 ("0" or "1": The optimization is considered fine.
## Other values indicate problems.)
```

The estimated regression coefficients and their 95% CIs on PD and AL are 0.0064 (-0.2050, 0.2177), and -0.0706 (-0.3883, 0.2471), respectively. The R^2 for predicting PD and AL are 0.0000, and 0.0433, respectively.

When there are multiple effect sizes, it is preferable to test the significance of all effect sizes simultaneously. We may formulate two nested models and compare them with the `anova()` function. The following analysis indicates that the likelihood ratio (LR) statistic for comparing both regression coefficients is $\chi^2(df = 2) = 0.3273, p = 0.8490$. Thus, the null hypothesis that both regression coefficients are zero is not rejected.

```
## Coefficients are fixed at 0 for both effect sizes
mult0 <- meta(y=cbind(PD,AL), v=cbind(var_PD,cov_PD_AL,var_AL),
              data=Berkey98, x=scale(pub_year,center=1979),
              model.name="Fixed at 0",
              coef.constraints=matrix(c("0","0"),nrow=2))
summary(mult0)

##
## Call:
## meta(y = cbind(PD, AL), v = cbind(var_PD, cov_PD_AL, var_AL),
##     x = scale(pub_year, center = 1979), data = Berkey98, coef.constraints
##         "0"), nrow = 2), model.name = "Fixed at 0")
```

```
##
## 95% confidence intervals: z statistic approximation
## Coefficients:
##              Estimate Std.Error    lbound    ubound z value Pr(>|z|)
## Intercept1  0.34484   0.05363  0.23972  0.44995    6.43  1.3e-10 ***
## Intercept2 -0.33794   0.08125 -0.49718 -0.17870   -4.16  3.2e-05 ***
## Tau2_1_1    0.00700   0.00905 -0.01074  0.02474    0.77     0.44
## Tau2_2_1    0.00946   0.00997 -0.01008  0.02900    0.95     0.34
## Tau2_2_2    0.02614   0.01774 -0.00863  0.06092    1.47     0.14
## ---
## Signif. codes:  0 '***' 0.001 '**' 0.01 '*' 0.05 '.' 0.1 ' ' 1
##
## Q statistic on the homogeneity of effect sizes: 128.2
## Degrees of freedom of the Q statistic: 8
## P value of the Q statistic: 0
##
## Explained variances (R2):
##                           y1   y2
## Tau2 (no predictor)    0.007 0.03
## Tau2 (with predictors) 0.007 0.03
## R2                     0.000 0.00
##
## Number of studies (or clusters): 5
## Number of observed statistics: 10
## Number of estimated parameters: 5
## Degrees of freedom: 5
## -2 log likelihood: -11.68
## OpenMx status1: 0 ("0" or "1": The optimization is considered fine.
## Other values indicate problems.)

## Compare two models with an LR statistic
anova(mult2, mult0)

##            base comparison ep minus2LL df    AIC diffLL diffdf     p
## 1 No constraint       <NA>  7   -12.01  3 -18.01     NA     NA    NA
## 2 No constraint Fixed at 0  5   -11.68  5 -21.68 0.3273      2 0.849
```

3.1.3 Multivariate fixed-effects model A multivariate fixed-effects meta-analysis is a special case of the random effects meta-analysis by fixing the variance component at a zero matrix. The pooled effect sizes with their 95% Wald CIs based on the fixed-effects model for PD and AL are 0.3072 (0.2512, 0.3632), and -0.3944 (-0.4310, -0.3578), respectively. It should be noted that the CIs on a fixed-effects model are usually shorter than those on a random-effects model when the heterogeneity is ignored in the analysis.

```
summary( meta(y=cbind(PD,AL), v=cbind(var_PD,cov_PD_AL,var_AL),
         RE.constraints=matrix(0,nrow=2,ncol=2), data=Berkey98,
         model.name="Fixed effects model") )

##
## Call:
```

```
## meta(y = cbind(PD, AL), v = cbind(var_PD, cov_PD_AL, var_AL),
##     data = Berkey98, RE.constraints = matrix(0, nrow = 2, ncol = 2),
##     model.name = "Fixed effects model")
##
## 95% confidence intervals: z statistic approximation
## Coefficients:
##              Estimate Std.Error  lbound  ubound z value Pr(>|z|)
## Intercept1    0.3072    0.0286  0.2512  0.3632    10.8   <2e-16 ***
## Intercept2   -0.3944    0.0186 -0.4309 -0.3578   -21.1   <2e-16 ***
## ---
## Signif. codes:  0 '***' 0.001 '**' 0.01 '*' 0.05 '.' 0.1 ' ' 1
##
## Q statistic on the homogeneity of effect sizes: 128.2
## Degrees of freedom of the Q statistic: 8
## P value of the Q statistic: 0
##
## Heterogeneity indices (based on the estimated Tau2):
##                               Estimate
## Intercept1: I2 (Q statistic)        0
## Intercept2: I2 (Q statistic)        0
##
## Number of studies (or clusters): 5
## Number of observed statistics: 10
## Number of estimated parameters: 2
## Degrees of freedom: 8
## -2 log likelihood: 90.88
## OpenMx status1: 0 ("0" or "1": The optimization is considered fine.
## Other values indicate problems.)
```

Although we may compare the fixed-effects model (without constraints) and the random-effects model (without any constraint) with a *LR* statistic, the *p* is too conservative. It is because it is testing on the boundary (e.g., **Stoel et al.**, 2006).

3.1.4 Plots of multivariate effect sizes If a multivariate meta-analysis is conducted, pairwise plots on the pooled effect sizes and their confidence ellipses can be obtained via the `plot()` function. This plot is a multivarite generalization of the forest plot in univariate meta-analysis. By default, 95% confidence intervals on the average effect sizes and confidence ellipses on the random effects are plotted (see **Cheung**, 2013). Figure 1 shows the average effect sizes of the `Berkey98` example. The black dots and the black dashed ellipses are the observed effect sizes and their 95% confidence ellipses in the primary studies. The blue square is the estimated average population effect sizes, while the red ellipse is the 95% confidence ellipse of estimated population average effect sizes. This is a multivariate generalization of the average effect size and its 95% confidence interval in univariate meta-analysis. The green ellipse is the 95% confidence ellipse of the random effects. Ninty-five percent of the studies with average population effect sizes falls inside this confidence ellipse in long run.

```
## Run the analysis again and save the object
my.fit <- meta(y=cbind(PD,AL),v=cbind(var_PD,cov_PD_AL,var_AL),
               data=Berkey98)

## No main title and label the axes
```

```
plot(my.fit, main="", axis.label=c("PD","AL"))
```

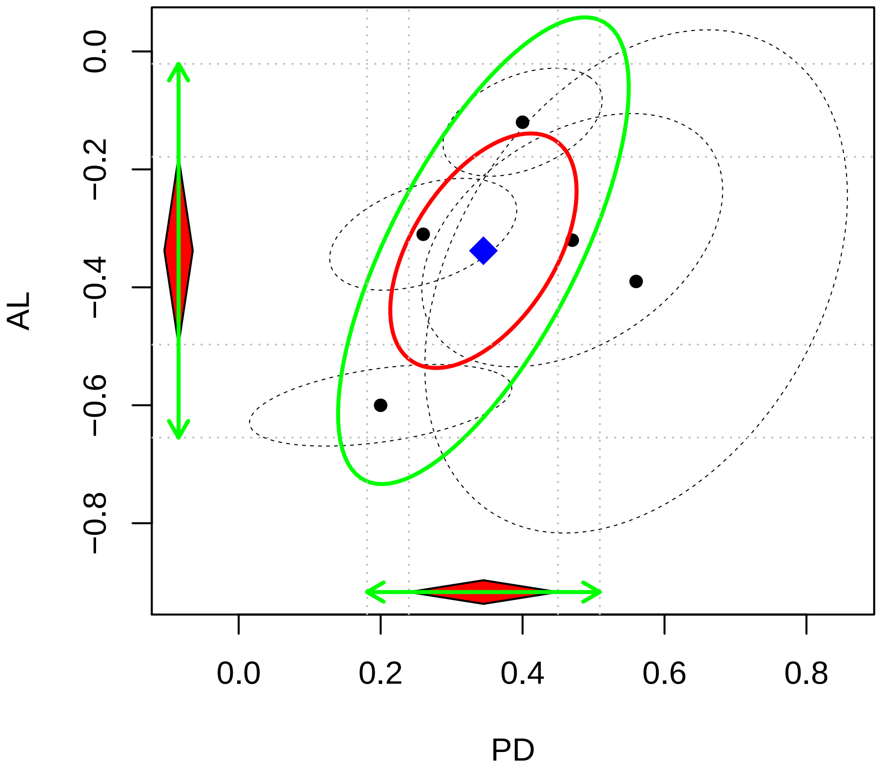

Figure 1. Plot of PD and AL

We may also combine the forest plots provided by the `metafor` package to provide more details on the individual effect sizes. Readers may understand both the univariate effects using the forest plot and the multivariate effects using the confidence ellipses. Figure 2 shows the confidence ellipses and the forest plots of the `Berkey98` example.

```
## Load the library for forest plots
library("metafor")

## Create extra panels for the forest plots
plot(my.fit, diag.panel=TRUE, main="Multivariate meta-analysis",
axis.label=c("PD", "AL"))

## Forest plot for PD
forest( rma(yi=PD, vi=var_PD, data=Berkey98) )
```

```
title("Forest plot of PD")

## Forest plot for AL
forest( rma(yi=AL, vi=var_AL, data=Berkey98) )
title("Forest plot of AL")
```

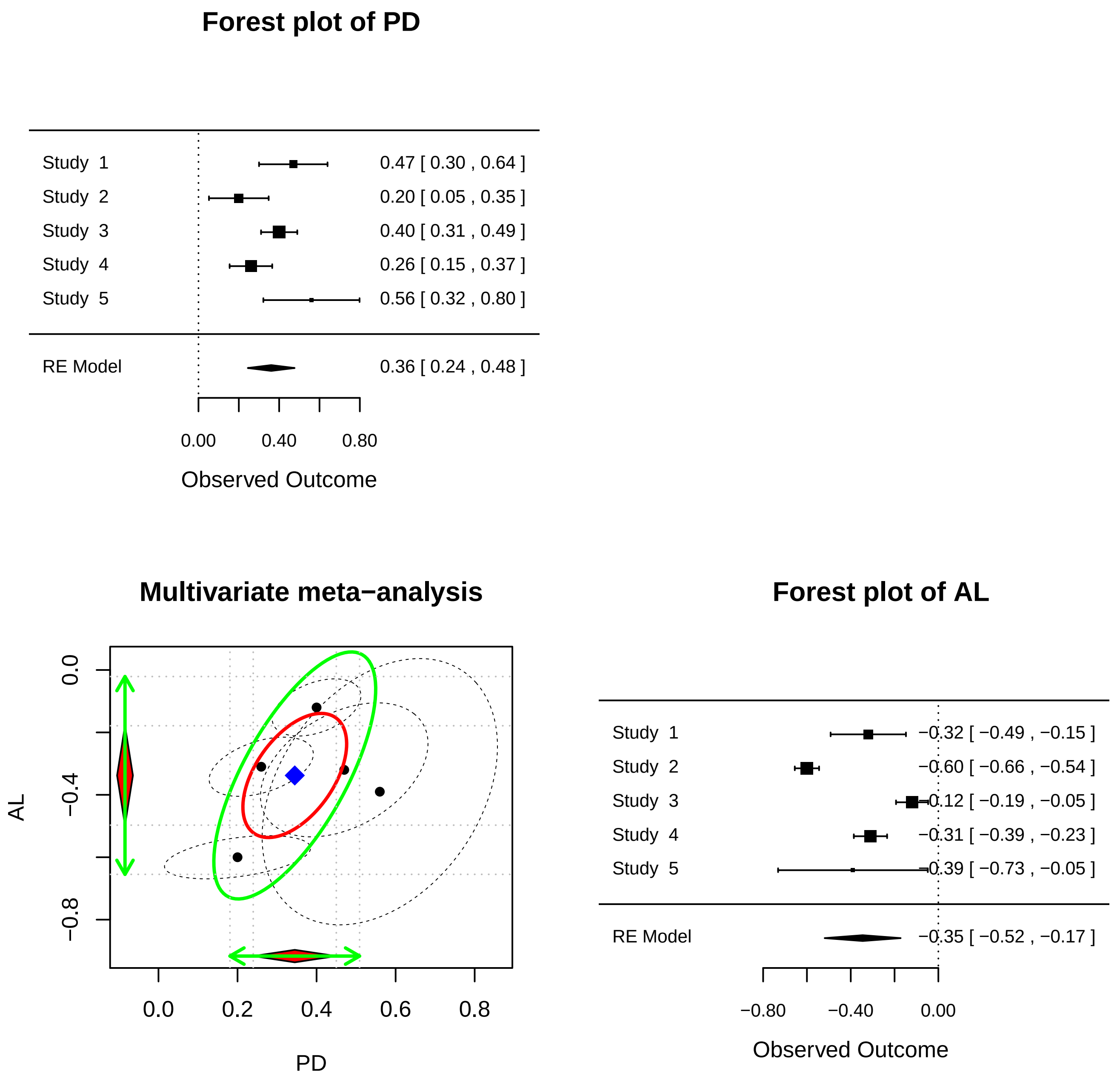

Figure 2. Plot of PD and AL with their forest plots

3.2 EXAMPLE 2

The second example was based on the sixteen studies reported by **Aloe et al.** (2014). These authors studied how the classroom management self-efficacy (CMSE) predicts the three dimensions of burnout. The effect sizes are the correlation coefficients between CMSE and emotional exhaustion (EE), depersonalization (DP), and (lowered) personal accomplishment (PA). Their sampling variances and covariances are labelled as `V_xx` and `C_xx_yy` in the data set where `xx` and `yy` are either `EE`, `DP` or `PA`.

```
## Show the first few cases
head(Aloe14)

##               Study Year    EE    DP   PA   V_EE   V_DP   V_PA C_EE_DP
## 1           Betoret 2009 -0.38 -0.32 0.62 0.0016 0.0018 0.0011  0.0005
## 2 Brouwers & Tomic 2000 -0.40 -0.39 0.56 0.0013 0.0009 0.0008  0.0006
## 3             Bumen 2010 -0.31 -0.34 0.48 0.0014 0.0014 0.0012  0.0007
## 4             Chang 2009 -0.32 -0.41 0.41 0.0021 0.0019 0.0019  0.0009
## 5              Durr 2008 -0.47 -0.54 0.71 0.0061 0.0063 0.0041  0.0032
## 6       Evers et al. 2002 -0.26 -0.31 0.39 0.0093 0.0067 0.0066  0.0028
##   C_EE_PA C_DP_PA Publication_type Percentage_females
## 1 -0.0002 -0.0003          Journal               0.70
## 2 -0.0004 -0.0004          Journal               0.26
## 3 -0.0003 -0.0004          Journal               0.69
## 4 -0.0010 -0.0011     Dissertation               0.79
## 5 -0.0010 -0.0012     Dissertation               0.82
## 6 -0.0015 -0.0045          Journal               0.23
##   Years_experience
## 1            10.08
## 2            21.25
## 3            18.14
## 4             2.58
## 5             2.71
## 6            22.14
```

3.2.1 Random-effects model We may conduct a multivariate random-effects with the following syntax. The average effect sizes and their *SE*s for `EE`, `DP`, and `PA` are -0.2805 (0.0302), -0.3262 (0.02823), and 0.4329 (0.0437), respectively. All the average effect sizes are statistically significant.

```
meta1 <- meta(y=cbind(EE,DP,PA),
              v=cbind(V_EE, C_EE_PA, C_EE_PA, V_DP, C_DP_PA, V_PA),
              data=Aloe14)
summary(meta1)

##
## Call:
## meta(y = cbind(EE, DP, PA), v = cbind(V_EE, C_EE_PA, C_EE_PA,
##     V_DP, C_DP_PA, V_PA), data = Aloe14)
##
## 95% confidence intervals: z statistic approximation
## Coefficients:
##             Estimate Std.Error    lbound    ubound z value Pr(>|z|)
```

```
## Intercept1 -0.280514  0.030159 -0.339626 -0.221403   -9.30   <2e-16
## Intercept2 -0.326245  0.028234 -0.381583 -0.270907  -11.55   <2e-16
## Intercept3  0.432889  0.043728  0.347185  0.518594    9.90   <2e-16
## Tau2_1_1    0.011266  0.005278  0.000921  0.021611    2.13    0.033
## Tau2_2_1    0.010430  0.004351  0.001902  0.018958    2.40    0.017
## Tau2_2_2    0.009763  0.004572  0.000802  0.018724    2.14    0.033
## Tau2_3_1   -0.016940  0.007157 -0.030967 -0.002912   -2.37    0.018
## Tau2_3_2   -0.014141  0.006434 -0.026751 -0.001530   -2.20    0.028
## Tau2_3_3    0.027268  0.011101  0.005510  0.049025    2.46    0.014
##
## Intercept1 ***
## Intercept2 ***
## Intercept3 ***
## Tau2_1_1   *
## Tau2_2_1   *
## Tau2_2_2   *
## Tau2_3_1   *
## Tau2_3_2   *
## Tau2_3_3   *
## ---
## Signif. codes:  0 '***' 0.001 '**' 0.01 '*' 0.05 '.' 0.1 ' ' 1
##
## Q statistic on the homogeneity of effect sizes: 91.44
## Degrees of freedom of the Q statistic: 45
## P value of the Q statistic: 5.266e-05
##
## Heterogeneity indices (based on the estimated Tau2):
##                              Estimate
## Intercept1: I2 (Q statistic)     0.80
## Intercept2: I2 (Q statistic)     0.81
## Intercept3: I2 (Q statistic)     0.93
##
## Number of studies (or clusters): 16
## Number of observed statistics: 48
## Number of estimated parameters: 9
## Degrees of freedom: 39
## -2 log likelihood: -96.61
## OpenMx status1: 0 ("0" or "1": The optimization is considered fine.
## Other values indicate problems.)
```

We may extract and arrange the variance component for ease of inspection.

```
## Extract the variance component of the random effects
( coef1 <- coef(meta1, select="random") )

##   Tau2_1_1   Tau2_2_1   Tau2_2_2   Tau2_3_1   Tau2_3_2   Tau2_3_3
##   0.011266   0.010430   0.009763  -0.016940  -0.014141   0.027268

## Convert it into a symmetrix matrix by row major
my.cov <- vec2symMat(coef1, byrow=TRUE)
```

```
## Add the dimensions for ease of interpretation
dimnames(my.cov) <- list( c("EE", "DP", "PA"),
                          c("EE", "DP", "PA") )
my.cov

##          EE        DP       PA
## EE  0.01127  0.010430 -0.01694
## DP  0.01043  0.009763 -0.01414
## PA -0.01694 -0.014141  0.02727

## Convert it into a correlation matrix
( cov2cor(my.cov) )

##         EE      DP      PA
## EE  1.0000  0.9945 -0.9665
## DP  0.9945  1.0000 -0.8667
## PA -0.9665 -0.8667  1.0000
```

The correlations among the random effects are extremely high. We may also visualize these correlations by the means of the confidence ellipses .

```
## Plot the multivariate effect sizes
plot(meta1, main="", axis.labels=c("EE", "DP", "PA"))
```

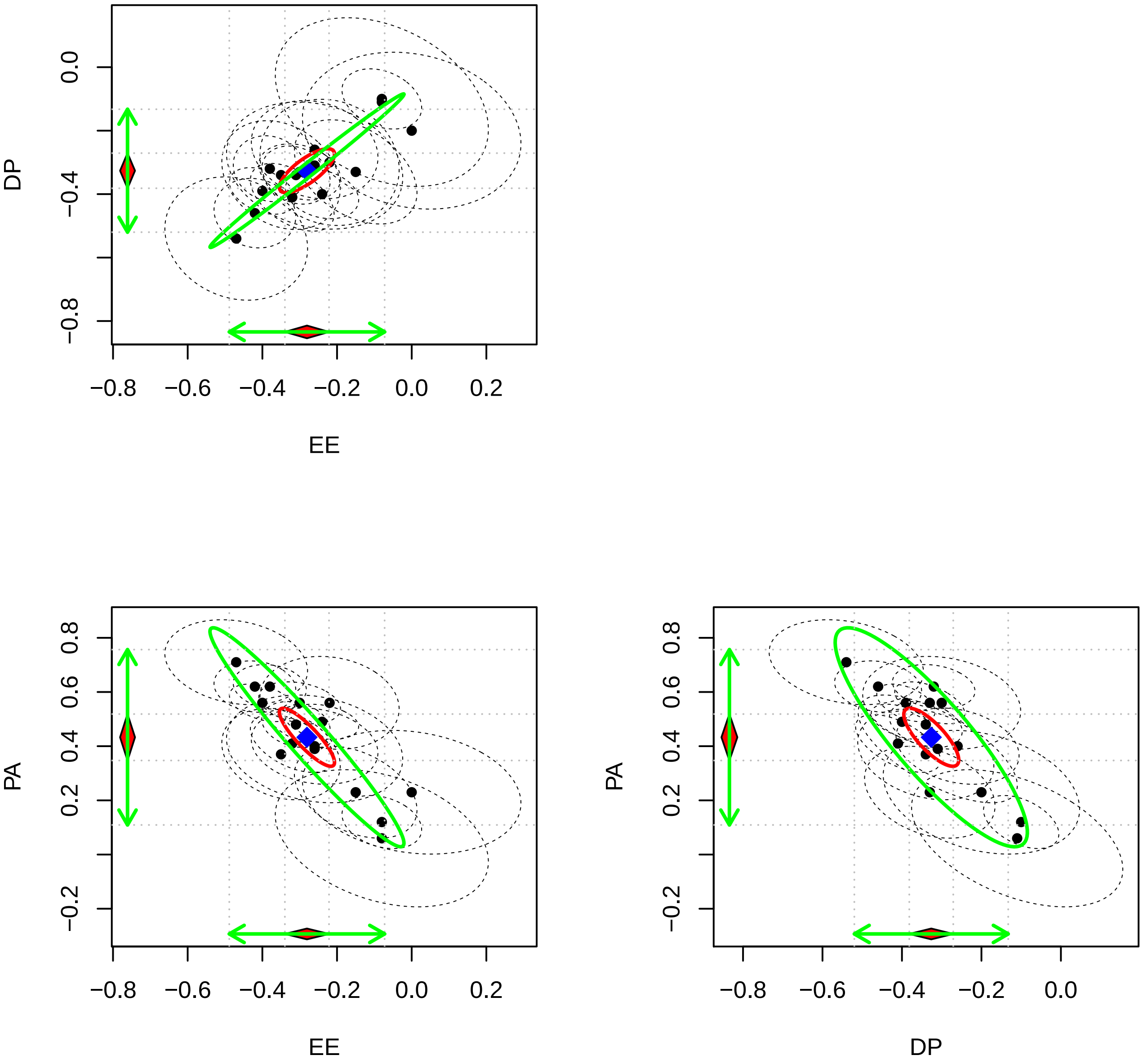

Figure 3. Plot of effect sizes and their confidence ellipses

3.2.2 Mixed-effects model **Aloe et al.** (2014) tested several potential moderators. One of them was whether or not the studies are published in peer-reviewed journals. We may replicate the analysis with the following code.

```
## Create a variable on journal
( journal <- ifelse(Aloe14$Publication_type=="Journal", 1, 0) )

##  [1] 1 1 1 0 0 1 1 1 1 0 0 0 1 1 1 0

meta2 <- meta(y=cbind(EE,DP,PA),
              v=cbind(V_EE, C_EE_PA, C_EE_PA, V_DP, C_DP_PA, V_PA),
              x=journal, data=Aloe14)
summary(meta2)

##
## Call:
## meta(y = cbind(EE, DP, PA), v = cbind(V_EE, C_EE_PA, C_EE_PA,
##     V_DP, C_DP_PA, V_PA), x = journal, data = Aloe14)
##
## 95% confidence intervals: z statistic approximation
## Coefficients:
##              Estimate Std.Error    lbound    ubound z value Pr(>|z|)
## Intercept1 -0.258899  0.053807 -0.364359 -0.153440   -4.81  1.5e-06
## Intercept2 -0.325399  0.050740 -0.424847 -0.225952   -6.41  1.4e-10
## Intercept3  0.392410  0.072997  0.249338  0.535483    5.38  7.6e-08
## Slope1_1   -0.033030  0.065110 -0.160642  0.094583   -0.51    0.612
## Slope2_1   -0.005488  0.061335 -0.125702  0.114726   -0.09    0.929
## Slope3_1    0.061996  0.090675 -0.115724  0.239715    0.68    0.494
## Tau2_1_1    0.011121  0.005194  0.000941  0.021301    2.14    0.032
## Tau2_2_1    0.010368  0.004296  0.001947  0.018788    2.41    0.016
## Tau2_2_2    0.009668  0.004523  0.000803  0.018534    2.14    0.033
## Tau2_3_1   -0.016672  0.007006 -0.030403 -0.002941   -2.38    0.017
## Tau2_3_2   -0.014203  0.006364 -0.026676 -0.001730   -2.23    0.026
## Tau2_3_3    0.026557  0.010799  0.005392  0.047723    2.46    0.014
##
## Intercept1 ***
## Intercept2 ***
## Intercept3 ***
## Slope1_1
## Slope2_1
## Slope3_1
## Tau2_1_1   *
## Tau2_2_1   *
## Tau2_2_2   *
## Tau2_3_1   *
## Tau2_3_2   *
## Tau2_3_3   *
## ---
## Signif. codes:  0 '***' 0.001 '**' 0.01 '*' 0.05 '.' 0.1 ' ' 1
##
## Q statistic on the homogeneity of effect sizes: 91.44
## Degrees of freedom of the Q statistic: 45
## P value of the Q statistic: 5.266e-05
##
## Explained variances (R2):
```

```
##                                 y1      y2   y3
## Tau2 (no predictor)     0.01127 0.00976 0.03
## Tau2 (with predictors)  0.01112 0.00967 0.03
## R2                      0.01289 0.00969 0.03
##
## Number of studies (or clusters): 16
## Number of observed statistics: 48
## Number of estimated parameters: 12
## Degrees of freedom: 36
## -2 log likelihood: -97.45
## OpenMx status1: 0 ("0" or "1": The optimization is considered fine.
## Other values indicate problems.)
```

The estimated slopes on predicting and their *SE*s for `EE`, `DP`, and `PA` are -0.0330 (0.0651), -0.0055 (0.0613), and 0.0620 (0.0907), respectively. All the slopes are non-significant. The R^2 for `EE`, `DP`, and `PA` are 0.01, 0.01, and 0.03, respectively.

We may also test the null hypothesis $\beta_{\text{EE}} = \beta_{\text{DP}} = \beta_{\text{PA}} = 0$ by comparing the models with and without the moderators with the following code. The $\chi^2(df = 3) = 0.8338, p = 0.8414$. Thus, the null hypothesis that both regression coefficients are zero is not rejected.

```
anova(meta2, meta1)

##                        base            comparison ep minus2LL df    AIC
## 1 Meta analysis with ML                      <NA> 12   -97.45 36 -169.4
## 2 Meta analysis with ML Meta analysis with ML  9   -96.61 39 -174.6
##   diffLL diffdf      p
## 1     NA     NA     NA
## 2 0.8338      3 0.8414
```

4 THREE-LEVEL META-ANALYSIS

This data set, reported by **Konstantopoulos** (2011) and **Cooper et al.** (2003), described fifty-six effect sizes clustered in 11 districts (`District`). The effect size is the standardized mean difference of the modified school calendar effectiveness. The `meta3()` function is used to fit three-level meta-analysis.

```
## Display the first few cases
head(Cooper03)

##   District Study     y     v Year
## 1       11     1 -0.18 0.118 1976
## 2       11     2 -0.22 0.118 1976
## 3       11     3  0.23 0.144 1976
## 4       11     4 -0.30 0.144 1976
## 5       12     5  0.13 0.014 1989
## 6       12     6 -0.26 0.014 1989
```

4.1 RANDOM-EFFECTS MODEL

The syntax of `meta3()` is very similar to that of `meta()` except that we need to specify the argument for `cluster`. For example,

```
summary( meta3(y=y, v=v, cluster=District, data=Cooper03) )

##
## Call:
## meta3(y = y, v = v, cluster = District, data = Cooper03)
##
## 95% confidence intervals: z statistic approximation
## Coefficients:
##             Estimate Std.Error   lbound   ubound z value Pr(>|z|)
## Intercept   0.18446   0.08054  0.02660  0.34231    2.29   0.0220 *
## Tau2_2      0.03286   0.01114  0.01103  0.05470    2.95   0.0032 **
## Tau2_3      0.05774   0.03074 -0.00252  0.11799    1.88   0.0604 .
## ---
## Signif. codes:  0 '***' 0.001 '**' 0.01 '*' 0.05 '.' 0.1 ' ' 1
##
## Q statistic on the homogeneity of effect sizes: 578.9
## Degrees of freedom of the Q statistic: 55
## P value of the Q statistic: 0
##
## Heterogeneity indices (based on the estimated Tau2):
##                               Estimate
## I2_2 (Typical v: Q statistic)     0.34
## I2_3 (Typical v: Q statistic)     0.60
##
## Number of studies (or clusters): 11
## Number of observed statistics: 56
## Number of estimated parameters: 3
## Degrees of freedom: 53
## -2 log likelihood: 16.79
## OpenMx status1: 0 ("0" or "1": The optimization is considered fine.
## Other values indicate problems.)
```

The analysis shows that the $Q(df = 55) = 578.9, p < 0.001$. The I^2 based on the Q statistic at level 2 and level 3 are 0.34, and 0.60, respectively. These indicate that the studies (level 2) and the cluster (level 3) explain about 34% and 60% percentages of the total variation. There is only 6% of the variation is due to sampling error. The average effect and its 95% CI under a random-effects model is 0.1845 (0.0266, 0.3423).

4.2 MIXED-EFFECTS MODEL

We use `Year` of publication as a moderator. To make the intercept more meaningful, we may center the predictor. The estimated coefficient (and its 95% Wald CI) of `Year` of publication in the following analysis is 0.0051 (-0.0116, 0.0218), which is not statistically significant. The R^2 at level 2 and level 3 are only 0.0000 and 0.0221, respectively.

```
summary( meta3(y=y, v=v, cluster=District,
               x=scale(Year, scale=FALSE), data=Cooper03) )

##
## Call:
## meta3(y = y, v = v, cluster = District, x = scale(Year, scale = FALSE),
##     data = Cooper03)
##
## 95% confidence intervals: z statistic approximation
## Coefficients:
##            Estimate Std.Error   lbound   ubound z value Pr(>|z|)
## Intercept   0.17803   0.08052  0.02021  0.33585    2.21   0.0270 *
## Slope_1     0.00507   0.00853 -0.01164  0.02179    0.60   0.5518
## Tau2_2      0.03294   0.01116  0.01106  0.05482    2.95   0.0032 **
## Tau2_3      0.05646   0.03003 -0.00240  0.11533    1.88   0.0601 .
## ---
## Signif. codes:  0 '***' 0.001 '**' 0.01 '*' 0.05 '.' 0.1 ' ' 1
##
## Q statistic on the homogeneity of effect sizes: 578.9
## Degrees of freedom of the Q statistic: 55
## P value of the Q statistic: 0
##
## Explained variances (R2):
##                          Level 2 Level 3
## Tau2 (no predictor)       0.0329    0.06
## Tau2 (with predictors)    0.0329    0.06
## R2                        0.0000    0.02
##
## Number of studies (or clusters): 11
## Number of observed statistics: 56
## Number of estimated parameters: 4
## Degrees of freedom: 52
## -2 log likelihood: 16.44
## OpenMx status1: 0 ("0" or "1": The optimization is considered fine.
## Other values indicate problems.)
```

5 META-ANALYTIC STRUCTURAL EQUATION MODELING

Two examples are used to illustrate how to use the TSSEM approach to fit structural equation models on the pooled correlation matrices.

5.1 EXAMPLE 1

Digman (1997) reported a second-order factor analysis on a five-factor model with 14 studies. He suggested that there were two second-order factors on the five-factor model: an **Alpha** factor for *agreeableness* `A`, *conscientiousness* `C`, and *emotional stability* `ES`, and a **Beta** factor for *extroversion* `E` and *intellect* `I`. We use the TSSEM approach to test the proposed model. This data set has been illustrated in several places (e.g., **Cheung and Chan**, 2005; **Cheung**, 2014, 2015). The correlation matrices and the

sample sizes are stored in `Digman97$data` and `Digman97$n`, respectively. We may display the first few cases of the data set by calling the following commands in R.

```
## Show the correlation matrices
head(Digman97$data)

## $`Digman 1 (1994)`
##          A     C   ES     E    I
## A    1.00  0.62 0.41 -0.48 0.00
## C    0.62  1.00 0.59 -0.10 0.35
## ES   0.41  0.59 1.00  0.27 0.41
## E   -0.48 -0.10 0.27  1.00 0.37
## I    0.00  0.35 0.41  0.37 1.00
##
## $`Digman 2 (1994)`
##          A    C   ES     E     I
## A    1.00 0.39 0.53 -0.30 -0.05
## C    0.39 1.00 0.59  0.07  0.44
## ES   0.53 0.59 1.00  0.09  0.22
## E   -0.30 0.07 0.09  1.00  0.45
## I   -0.05 0.44 0.22  0.45  1.00
##
## $`Digman 3 (1963c)`
##         A     C   ES     E    I
## A   1.00  0.65 0.35  0.25 0.14
## C   0.65  1.00 0.37 -0.10 0.33
## ES  0.35  0.37 1.00  0.24 0.41
## E   0.25 -0.10 0.24  1.00 0.41
## I   0.14  0.33 0.41  0.41 1.00
##
## $`Digman & Takemoto-Chock (1981b)`
##          A     C   ES     E     I
## A    1.00  0.65 0.70 -0.26 -0.03
## C    0.65  1.00 0.71 -0.16  0.24
## ES   0.70  0.71 1.00  0.01  0.11
## E   -0.26 -0.16 0.01  1.00  0.66
## I   -0.03  0.24 0.11  0.66  1.00
##
## $`Graziano & Ward (1992)`
##         A    C   ES    E    I
## A   1.00 0.64 0.35 0.29 0.22
## C   0.64 1.00 0.27 0.16 0.22
## ES  0.35 0.27 1.00 0.32 0.36
## E   0.29 0.16 0.32 1.00 0.53
## I   0.22 0.22 0.36 0.53 1.00
##
## $`Yik & Bond (1993)`
##         A    C   ES    E    I
## A   1.00 0.66 0.57 0.35 0.38
## C   0.66 1.00 0.45 0.20 0.31
## ES  0.57 0.45 1.00 0.49 0.31
## E   0.35 0.20 0.49 1.00 0.59
```

```
## I  0.38 0.31 0.31 0.59 1.00

## Show the sample sizes
head(Digman97$n)

## [1] 102 149 334 162  91 656
```

5.1.1 Fixed-effects model

Stage 1 analysis The `tssem1()` function is used to pool the correlation matrices with a fixed-effects model in the first stage of the analysis by specifying `method="FEM"` in the argument:

```
fixed1 <- tssem1(Digman97$data, Digman97$n, method = "FEM")
summary(fixed1)

##
## Call:
## tssem1FEM(my.df = my.df, n = n, cor.analysis = cor.analysis,
##     model.name = model.name, cluster = cluster, suppressWarnings = suppres
##     silent = silent, run = run)
##
## Coefficients:
##        Estimate Std.Error z value Pr(>|z|)
## S[1,2]   0.3631    0.0134   27.12  < 2e-16 ***
## S[1,3]   0.3902    0.0129   30.24  < 2e-16 ***
## S[1,4]   0.1038    0.0151    6.88  5.8e-12 ***
## S[1,5]   0.0922    0.0151    6.12  9.3e-10 ***
## S[2,3]   0.4160    0.0125   33.17  < 2e-16 ***
## S[2,4]   0.1352    0.0148    9.14  < 2e-16 ***
## S[2,5]   0.1412    0.0149    9.48  < 2e-16 ***
## S[3,4]   0.2445    0.0142   17.25  < 2e-16 ***
## S[3,5]   0.1382    0.0149    9.30  < 2e-16 ***
## S[4,5]   0.4245    0.0124   34.25  < 2e-16 ***
## ---
## Signif. codes:  0 '***' 0.001 '**' 0.01 '*' 0.05 '.' 0.1 ' ' 1
##
## Goodness-of-fit indices:
##                                       Value
## Sample size                         4496.00
## Chi-square of target model          1499.73
## DF of target model                   130.00
## p value of target model                0.00
## Chi-square of independence model    4454.60
## DF of independence model             140.00
## RMSEA                                  0.18
## SRMR                                   0.16
## TLI                                    0.66
## CFI                                    0.68
## AIC                                 1239.73
## BIC                                  406.31
```

```
## OpenMx status1: 0 ("0" or "1": The optimization is considered fine.
## Other values indicate problems.)
```

The fit indices for testing the homogeneity of the correlation matrices in the Stage 1 analysis are $\chi^2(df = 130, N = 4,496) = 1,499.73, p < 0.001$, CFI=0.68, TLI=0.66, SRMR=0.16, and RMSEA=0.18. These value indicate that it is not reasonable to assume that the correlation matrices are homogeneous. Rather, it would be more appropriate to employ a random-effects model that will be illustrated later. As an illustration, however, we continue to fit the stage 2 model even though the homogeneity assumption of the correlation matrices is questionable.

We may also extract the pooled correlation matrix by the following command.

```
coef(fixed1)

##          x1     x2     x3     x4      x5
## x1 1.00000 0.3631 0.3902 0.1038 0.09225
## x2 0.36312 1.0000 0.4160 0.1352 0.14121
## x3 0.39018 0.4160 1.0000 0.2445 0.13817
## x4 0.10375 0.1352 0.2445 1.0000 0.42451
## x5 0.09225 0.1412 0.1382 0.4245 1.00000
```

Stage 2 analysis The `tssem2()` function is then used to fit a factor analytic model on the pooled correlation matrix with the inverse of its asymptotic covariance matrix as the weight matrix. The structural model in the stage 2 analysis is specified via the reticular action model (RAM) formulation (**McArdle and McDonald**, 1984). Structural models are specified via three matrices. **A** and **S** are used to specify the asymmetric paths and the symmetric variance covariance matrices, respectively. **A** denotes the asymmetric paths, such as the regression coefficients and the factor loadings among the variables, with a_{ij} in **A** representing the regression coefficient from variable j to variable i. **S** is a symmetric matrix representing the variances and covariances of the variables. It is used to specify the double arrows in path diagrams. The diagonal elements represent the variances of the variables. If the variables are independent variables, the corresponding diagonals in **S** denote the variances; otherwise, the corresponding diagonals in **S** represent the residuals of the dependent variables. The off-diagonals in **S** represent the covariances of the variables. **F** is a selection matrix used to filter observed variables. The following syntax specifies the **A** matrix:

```
## Factor loadings
Lambda <-  matrix(c(".3*Alpha_A", ".3*Alpha_C", ".3*Alpha_ES",
                    rep(0,5),".3*Beta_E", ".3*Beta_I"),
                  ncol = 2, nrow = 5)

## It is easier to create A this way since there are lots of 0
A1 <- rbind(cbind(matrix(0,ncol=5,nrow=5), Lambda),
           matrix(0,ncol=7,nrow=2))

## This step is not necessary but is helpful in inspecting the content of A1
dimnames(A1) <- list(c("A", "C", "ES", "E", "I", "Alpha", "Beta"),
                     c("A", "C", "ES", "E", "I", "Alpha", "Beta"))
## Display the content of A1
A1

##       A   C   ES  E   I   Alpha          Beta
```

```
## A      "0" "0" "0" "0" "0" ".3*Alpha_A"  "0"
## C      "0" "0" "0" "0" "0" ".3*Alpha_C"  "0"
## ES     "0" "0" "0" "0" "0" ".3*Alpha_ES" "0"
## E      "0" "0" "0" "0" "0" "0"           ".3*Beta_E"
## I      "0" "0" "0" "0" "0" "0"           ".3*Beta_I"
## Alpha  "0" "0" "0" "0" "0" "0"           "0"
## Beta   "0" "0" "0" "0" "0" "0"           "0"
```

The above output shows the **A1** matrix. `Alpha_A` is the label of the factor loading from **Alpha** to **A**, while ”0.3” is the starting value. When the labels are the same, the parameters are constrained equally. The values of ”0” mean that these factor loadings are fixed at 0. The following syntax specifies the **S** matrix:

```
## Covariance matrix among the latent factors
Phi <- matrix(c(1, "0.3*cor", "0.3*cor",1), ncol=2, nrow=2)
## Error variances among the errors
Psi <- Diag(c(".2*e1", ".2*e2", ".2*e3", ".2*e4", ".2*e5"))

## Combine them to create the S matrix
S1 <- bdiagMat(list(Psi, Phi))

## This step is not necessary but is helpful in inspecting the content of S1.
dimnames(S1) <- list(c("A", "C", "ES", "E", "I", "Alpha", "Beta"),
                     c("A", "C", "ES", "E", "I", "Alpha", "Beta"))
S1

##        A        C        ES       E        I        Alpha     Beta
## A      ".2*e1" "0"      "0"      "0"      "0"      "0"       "0"
## C      "0"     ".2*e2" "0"      "0"      "0"      "0"       "0"
## ES     "0"     "0"      ".2*e3" "0"      "0"      "0"       "0"
## E      "0"     "0"      "0"      ".2*e4" "0"      "0"       "0"
## I      "0"     "0"      "0"      "0"      ".2*e5" "0"       "0"
## Alpha  "0"     "0"      "0"      "0"      "0"      "1"       "0.3*cor"
## Beta   "0"     "0"      "0"      "0"      "0"      "0.3*cor" "1"
```

The following syntax specifies the **F** matrix:

```
## The first 5 variables are observed, whereas the last 2 are latent.
F1 <- create.Fmatrix(c(1, 1, 1, 1, 1, 0, 0), as.mxMatrix=FALSE)
## This step is not necessary but is helpful in inspecting the content of F1.
dimnames(F1) <- list(c("A", "C", "ES", "E", "I"),
                     c("A", "C", "ES", "E", "I", "Alpha", "Beta"))
F1

##     A C ES E I Alpha Beta
## A   1 0  0 0 0     0    0
## C   0 1  0 0 0     0    0
## ES  0 0  1 0 0     0    0
## E   0 0  0 1 0     0    0
## I   0 0  0 0 1     0    0
```

We may then fit the structural model via the `tssem2()` command:

```
fixed2 <- tssem2(fixed1, Amatrix=A1, Smatrix=S1, Fmatrix=F1,
                 model.name="Digman97 FEM")
summary(fixed2)

##
## Call:
## wls(Cov = pooledS, asyCov = tssem1.obj$acovS, n = sum(tssem1.obj$n),
##     Amatrix = Amatrix, Smatrix = Smatrix, Fmatrix = Fmatrix,
##     diag.constraints = diag.constraints, cor.analysis = cor.analysis,
##     intervals.type = intervals.type, mx.algebras = mx.algebras,
##     model.name = model.name, suppressWarnings = suppressWarnings,
##     silent = silent, run = run)
##
## 95% confidence intervals: z statistic approximation
## Coefficients:
##          Estimate Std.Error lbound ubound z value Pr(>|z|)
## Alpha_A    0.5626    0.0154 0.5324 0.5928    36.5   <2e-16 ***
## Alpha_C    0.6051    0.0154 0.5750 0.6352    39.4   <2e-16 ***
## Alpha_ES   0.7191    0.0157 0.6883 0.7499    45.8   <2e-16 ***
## Beta_E     0.7820    0.0343 0.7147 0.8493    22.8   <2e-16 ***
## Beta_I     0.5509    0.0261 0.4998 0.6020    21.1   <2e-16 ***
## cor        0.3626    0.0224 0.3187 0.4066    16.2   <2e-16 ***
## ---
## Signif. codes:  0 '***' 0.001 '**' 0.01 '*' 0.05 '.' 0.1 ' ' 1
##
## Goodness-of-fit indices:
##                                               Value
## Sample size                                 4496.00
## Chi-square of target model                    65.06
## DF of target model                             4.00
## p value of target model                        0.00
## Number of constraints imposed on "Smatrix"     0.00
## DF manually adjusted                           0.00
## Chi-square of independence model            3100.24
## DF of independence model                      10.00
## RMSEA                                          0.06
## SRMR                                           0.03
## TLI                                            0.95
## CFI                                            0.98
## AIC                                           57.06
## BIC                                           31.42
## OpenMx status1: 0 ("0" or "1": The optimization is considered fine.
## Other values indicate problems.)
```

The fit indices on the Stage 2 structural model are $\chi^2(df = 4, N = 4,496) = 65.06, p < 0.001$, CFI=0.98, TLI=0.95, SRMR=0.03, and RMSEA=0.06. Although the goodness-of-fit indices look good, we should be cautious when interpreting them because of the poor goodness-of-fit indices in the Stage 1 analysis.

5.1.2 *Random-effects model*

Stage 1 analysis The random-effects TSSEM may be requested by specifying the `method="REM"` argument in `tssem1()`. By default (`RE.type="Symm"`), a positive definite symmetric covariance matrix among the random effects is used. For practical reasons, such as an insufficient number of studies, it may not be feasible to estimate the full variance components of the random effects. A diagonal matrix of the random effects may be specified by using `RE.type="Diag"`. Researchers may also specify `RE.type="Zero"`. Since the variance component of the random effects is zero, the model becomes a fixed-effects model. This model is equivalent to the Generalized Least Squares (GLS) approach proposed by **Becker** (1992).

```
random1 <- tssem1(Digman97$data, Digman97$n, method="REM", RE.type="Diag")
summary(random1)

##
## Call:
## meta(y = ES, v = acovR, RE.constraints = Diag(x = paste(RE.startvalues,
##     "*Tau2_", 1:no.es, "_", 1:no.es, sep = "")), RE.lbound = RE.lbound,
##     I2 = I2, model.name = model.name, suppressWarnings = TRUE,
##     silent = silent, run = run)
##
## 95% confidence intervals: z statistic approximation
## Coefficients:
##              Estimate Std.Error    lbound    ubound z value Pr(>|z|)
## Intercept1   3.95e-01  5.42e-02  2.88e-01  5.01e-01    7.28  3.4e-13
## Intercept2   4.40e-01  4.13e-02  3.59e-01  5.21e-01   10.67  < 2e-16
## Intercept3   5.45e-02  6.17e-02 -6.64e-02  1.76e-01    0.88   0.3768
## Intercept4   9.87e-02  4.62e-02  8.08e-03  1.89e-01    2.13   0.0328
## Intercept5   4.30e-01  4.02e-02  3.51e-01  5.08e-01   10.70  < 2e-16
## Intercept6   1.29e-01  4.08e-02  4.85e-02  2.09e-01    3.15   0.0016
## Intercept7   2.05e-01  4.96e-02  1.08e-01  3.02e-01    4.14  3.5e-05
## Intercept8   2.40e-01  3.19e-02  1.77e-01  3.03e-01    7.52  5.7e-14
## Intercept9   1.89e-01  4.30e-02  1.05e-01  2.73e-01    4.40  1.1e-05
## Intercept10  4.44e-01  3.25e-02  3.80e-01  5.08e-01   13.65  < 2e-16
## Tau2_1_1     3.72e-02  1.50e-02  7.81e-03  6.66e-02    2.48   0.0131
## Tau2_2_2     2.03e-02  8.43e-03  3.77e-03  3.68e-02    2.41   0.0161
## Tau2_3_3     4.82e-02  1.97e-02  9.56e-03  8.69e-02    2.44   0.0145
## Tau2_4_4     2.46e-02  1.06e-02  3.79e-03  4.54e-02    2.32   0.0205
## Tau2_5_5     1.87e-02  8.25e-03  2.56e-03  3.49e-02    2.27   0.0232
## Tau2_6_6     1.83e-02  8.79e-03  1.03e-03  3.55e-02    2.08   0.0378
## Tau2_7_7     2.94e-02  1.23e-02  5.39e-03  5.35e-02    2.40   0.0164
## Tau2_8_8     9.65e-03  4.88e-03  8.17e-05  1.92e-02    1.98   0.0481
## Tau2_9_9     2.09e-02  9.13e-03  3.04e-03  3.88e-02    2.29   0.0218
## Tau2_10_10   1.12e-02  5.05e-03  1.26e-03  2.10e-02    2.21   0.0271
##
## Intercept1  ***
## Intercept2  ***
## Intercept3
## Intercept4  *
## Intercept5  ***
## Intercept6  **
## Intercept7  ***
```

```
## Intercept8  ***
## Intercept9  ***
## Intercept10 ***
## Tau2_1_1    *
## Tau2_2_2    *
## Tau2_3_3    *
## Tau2_4_4    *
## Tau2_5_5    *
## Tau2_6_6    *
## Tau2_7_7    *
## Tau2_8_8    *
## Tau2_9_9    *
## Tau2_10_10  *
## ---
## Signif. codes:  0 '***' 0.001 '**' 0.01 '*' 0.05 '.' 0.1 ' ' 1
##
## Q statistic on the homogeneity of effect sizes: 2381
## Degrees of freedom of the Q statistic: 130
## P value of the Q statistic: 0
##
## Heterogeneity indices (based on the estimated Tau2):
##                               Estimate
## Intercept1: I2 (Q statistic)      0.95
## Intercept2: I2 (Q statistic)      0.91
## Intercept3: I2 (Q statistic)      0.94
## Intercept4: I2 (Q statistic)      0.89
## Intercept5: I2 (Q statistic)      0.90
## Intercept6: I2 (Q statistic)      0.85
## Intercept7: I2 (Q statistic)      0.91
## Intercept8: I2 (Q statistic)      0.77
## Intercept9: I2 (Q statistic)      0.87
## Intercept10: I2 (Q statistic)     0.84
##
## Number of studies (or clusters): 14
## Number of observed statistics: 140
## Number of estimated parameters: 20
## Degrees of freedom: 120
## -2 log likelihood: -110.8
## OpenMx status1: 0 ("0" or "1": The optimization is considered fine.
## Other values indicate problems.)
```

The I^2 indicates the heterogeneity of the correlation coefficients. For example, the following analysis shows that the I^2 based on the Q statistic varies from 0.84 to 0.95, indicating a high degree of heterogeneity among the correlation elements. There is no goodness-of-fit indices for the random-effects TSSEM since it is usually based on a saturated model of mean vectors of fixed effects and variance components of random effects in a multivariate random-effects meta-analysis.

If we want to extract the estimated average correlation matrix in matrix form, we may use the following command.

```
## Select the fixed effects and convert it into a correlation matrix
vec2symMat( coef(random1, select="fixed"), diag=FALSE )

##         [,1]   [,2]   [,3]    [,4]    [,5]
## [1,] 1.00000 0.3946 0.4401 0.05454 0.09867
## [2,] 0.39465 1.0000 0.4297 0.12851 0.20526
## [3,] 0.44009 0.4297 1.0000 0.23994 0.18910
## [4,] 0.05454 0.1285 0.2399 1.00000 0.44413
## [5,] 0.09867 0.2053 0.1891 0.44413 1.00000
```

Stage 2 analysis The Stage 2 analysis is conducted as usual via the `tssem2()` function. This functions automatically handles whether a fixed- or a random-effects model is used in the stage 1 analysis.

```
random2 <- tssem2(random1, Amatrix=A1, Smatrix=S1, Fmatrix=F1)
summary(random2)

##
## Call:
## wls(Cov = pooledS, asyCov = asyCov, n = tssem1.obj$total.n, Amatrix = Amat
##     Smatrix = Smatrix, Fmatrix = Fmatrix, diag.constraints = diag.constrai
##     cor.analysis = cor.analysis, intervals.type = intervals.type,
##     mx.algebras = mx.algebras, model.name = model.name, suppressWarnings =
##     silent = silent, run = run)
##
## 95% confidence intervals: z statistic approximation
## Coefficients:
##          Estimate Std.Error lbound ubound z value Pr(>|z|)
## Alpha_A    0.5726    0.0516 0.4714 0.6737   11.10  < 2e-16 ***
## Alpha_C    0.5901    0.0518 0.4885 0.6917   11.38  < 2e-16 ***
## Alpha_ES   0.7705    0.0610 0.6508 0.8901   12.62  < 2e-16 ***
## Beta_E     0.6934    0.0748 0.5468 0.8400    9.27  < 2e-16 ***
## Beta_I     0.6401    0.0689 0.5052 0.7751    9.30  < 2e-16 ***
## cor        0.3937    0.0476 0.3004 0.4869    8.28  2.2e-16 ***
## ---
## Signif. codes:  0 '***' 0.001 '**' 0.01 '*' 0.05 '.' 0.1 ' ' 1
##
## Goodness-of-fit indices:
##                                               Value
## Sample size                                 4496.00
## Chi-square of target model                     8.51
## DF of target model                             4.00
## p value of target model                        0.07
## Number of constraints imposed on "Smatrix"     0.00
## DF manually adjusted                           0.00
## Chi-square of independence model            514.56
## DF of independence model                      10.00
## RMSEA                                          0.02
## SRMR                                           0.05
## TLI                                            0.98
## CFI                                            0.99
```

```
## AIC                                                0.51
## BIC                                              -25.13
## OpenMx status1: 0 ("0" or "1": The optimization is considered fine.
## Other values indicate problems.)
```

The fit indices on the Stage 2 structural model are $\chi^2(df = 4, N = 4,496) = 8.51, p < 0.001$, CFI=0.99, TLI=0.98, SRMR=0.05, and RMSEA=0.02. This indicates that the model fits the data quite well. The factor loadings on the **Alpha** factor are 0.5726, 0.5901, and 0.7705, while the factor loadings on the **Beta** factor are 0.6934, and 0.6401. The factor correlation between these two factors is 0.3937. All of these estimates are statistically significant.

We may check whether the parameters are correctly labelled by displaying the model graphically. This helps us to check whether the theoretical model is the same as the fitted one.

```
## Library to plot the model
library("semPlot")

## Convert the model to semPlotModel object
## latNames: Names of the latent variables
my.plot <- meta2semPlot(random2, latNames=c("Alpha","Beta"))

## Plot the model with parameter labels
semPaths(my.plot, whatLabels="path", nCharEdges=10, nCharNodes=10,
         color="yellow", edge.label.cex=0.8)
```

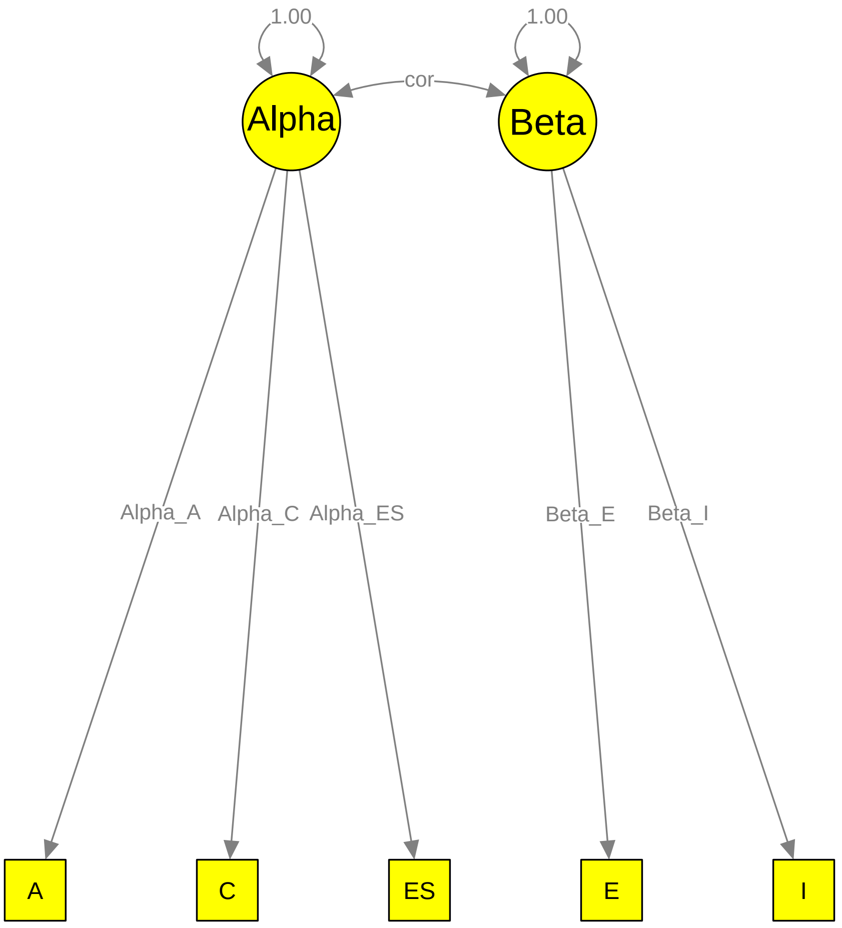

More importantly, we may plot the parameter estimates by the following command.

```
## Plot the parameter estimates
semPaths(my.plot, whatLabels="est", nCharNodes=10, color="green",
         edge.label.cex=1.2)
```

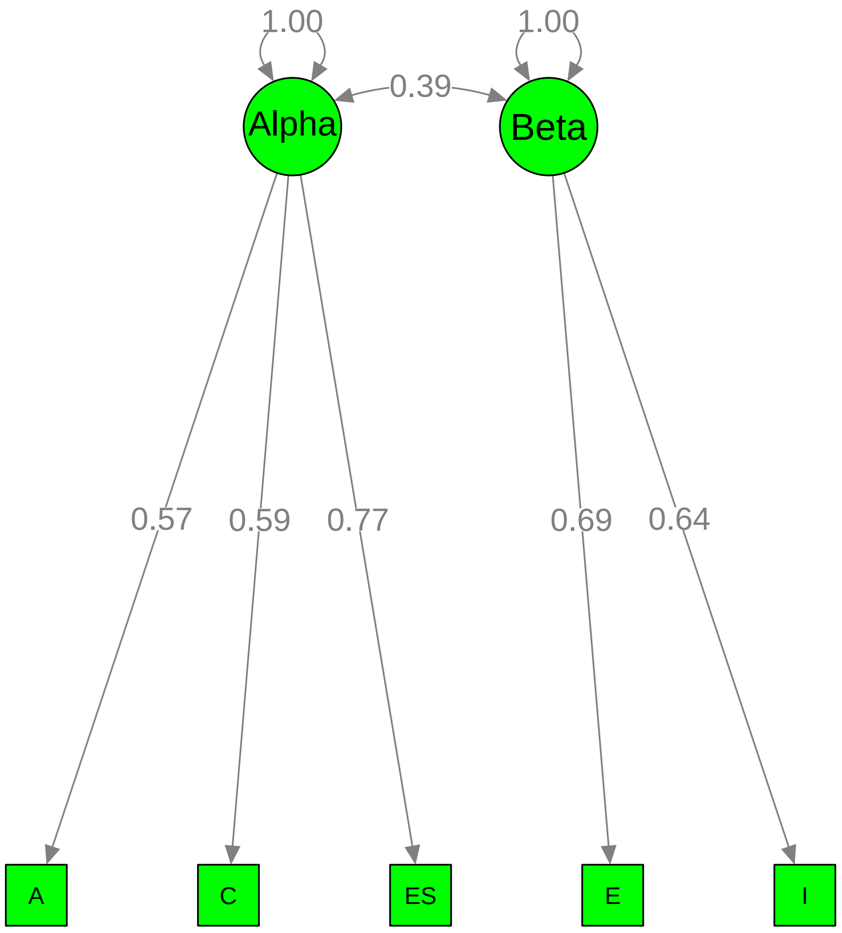

5.2 EXAMPLE 2

This dataset was based on **Becker** (2009); **Craft et al.** (2003). It includes ten studies of correlation matrices among *Performance* `Per`, *Cognitive* `Cog`, *Somatic* `SO`, and *Self confidence* `SC`. The dependent variable is *Performance*, while the other variables are either independent variables or mediators.

```
## Display the first few cases of the data
head(Becker09$data)

## $`1`
##                  Performance Cognitive Somatic Self confidence
## Performance             1.00     -0.55   -0.48            0.66
## Cognitive              -0.55      1.00    0.47           -0.38
## Somatic                -0.48      0.47    1.00           -0.46
```

```
## Self confidence        0.66      -0.38   -0.46             1.00
##
## $`3`
##                  Performance Cognitive Somatic Self confidence
## Performance             1.00      0.53   -0.12             0.03
## Cognitive               0.53      1.00    0.52            -0.48
## Somatic                -0.12      0.52    1.00            -0.40
## Self confidence         0.03     -0.48   -0.40             1.00
##
## $`6`
##                  Performance Cognitive Somatic Self confidence
## Performance             1.00      0.44    0.46               NA
## Cognitive               0.44      1.00    0.67               NA
## Somatic                 0.46      0.67    1.00               NA
## Self confidence           NA        NA      NA               NA
##
## $`10`
##                  Performance Cognitive Somatic Self confidence
## Performance             1.00     -0.39   -0.17             0.19
## Cognitive              -0.39      1.00    0.21            -0.54
## Somatic                -0.17      0.21    1.00            -0.43
## Self confidence         0.19     -0.54   -0.43             1.00
##
## $`17`
##                  Performance Cognitive Somatic Self confidence
## Performance             1.00       0.1    0.31            -0.17
## Cognitive               0.10       1.0      NA               NA
## Somatic                 0.31        NA      NA               NA
## Self confidence        -0.17        NA      NA               NA
##
## $`22`
##                  Performance Cognitive Somatic Self confidence
## Performance             1.00      0.23    0.08             0.51
## Cognitive               0.23      1.00    0.45            -0.29
## Somatic                 0.08      0.45    1.00            -0.44
## Self confidence         0.51     -0.29   -0.44             1.00

## Display the sample sizes
Becker09$n

##  [1] 142  37  16  14  45 100  51 128  70  30
```

5.2.1 Fixed-effects model

Stage 1 analysis We may conduct the first stage analysis with a fixed-effects TSSEM with the following syntax. The fit indices for testing the homogeneity of the correlation matrices in the Stage 1 analysis are $\chi^2(df = 46, N = 633) = 208.94, p < 0.001$, CFI=0.72, TLI=0.68, SRMR=0.20, and RMSEA=0.24. These value indicate that it is not reasonable to assume that the correlation matrices are homogeneous.

```
## First stage analysis
fixed1 <- tssem1(Becker09$data, Becker09$n, method="FEM")
summary(fixed1)

##
## Call:
## tssem1FEM(my.df = my.df, n = n, cor.analysis = cor.analysis,
##     model.name = model.name, cluster = cluster, suppressWarnings = suppres
##     silent = silent, run = run)
##
## Coefficients:
##        Estimate Std.Error z value Pr(>|z|)
## S[1,2]  -0.0685    0.0423   -1.62  0.10556
## S[1,3]  -0.1581    0.0413   -3.83  0.00013 ***
## S[1,4]   0.3715    0.0373    9.95  < 2e-16 ***
## S[2,3]   0.5262    0.0302   17.42  < 2e-16 ***
## S[2,4]  -0.4135    0.0352  -11.76  < 2e-16 ***
## S[3,4]  -0.4164    0.0350  -11.89  < 2e-16 ***
## ---
## Signif. codes:  0 '***' 0.001 '**' 0.01 '*' 0.05 '.' 0.1 ' ' 1
##
## Goodness-of-fit indices:
##                                     Value
## Sample size                        633.00
## Chi-square of target model         208.94
## DF of target model                  46.00
## p value of target model              0.00
## Chi-square of independence model  628.76
## DF of independence model            52.00
## RMSEA                                0.24
## SRMR                                 0.20
## TLI                                  0.68
## CFI                                  0.72
## AIC                                116.94
## BIC                                -87.78
## OpenMx status1: 0 ("0" or "1": The optimization is considered fine.
## Other values indicate problems.)
```

Stage 2 analysis A random-effects model is preferred for this data set. As an illustration, we also fit the structural equation model in the stage two analysis. Since there are ”mediators” in the model, the argument `diag.constraints=TRUE` must be specified. As there is no `SE` with the specification of the argument `diag.constraints=TRUE`, we may request the LBCI by specifying `intervals.type="LB"`.

```
## Regression coefficients
A1 <- create.mxMatrix(c(0, "0.1*Cog2Per", "0.1*SO2Per", "0.1*SC2Per",
                        0, 0, 0, 0,
                        0, 0, 0, 0,
                        0, "0.1*Cog2SC", "0.1*SO2SC",0),
                      type="Full", byrow=TRUE, ncol=4, nrow=4,
                      as.mxMatrix=FALSE)
```

```
## This step is not necessary but it is useful for inspecting the model.
dimnames(A1)[[1]] <- dimnames(A1)[[2]] <- c("Per","Cog","SO","SC")

A1

##     Per Cog            SO            SC
## Per "0" "0.1*Cog2Per" "0.1*SO2Per" "0.1*SC2Per"
## Cog "0" "0"            "0"           "0"
## SO  "0" "0"            "0"           "0"
## SC  "0" "0.1*Cog2SC"  "0.1*SO2SC"   "0"

## Covariance matrix among the variables
S1 <- create.mxMatrix(c("0.1*var_Per",
                        0, 1,
                        0, "0.1*cor", 1,
                        0, 0, 0, "0.1*var_SC"), byrow=TRUE, type="Symm",
                      as.mxMatrix=FALSE)

## This step is not necessary but it is useful for inspecting the model.
dimnames(S1)[[1]] <- dimnames(S1)[[2]] <- c("Per","Cog","SO","SC")
S1

##     Per           Cog       SO        SC
## Per "0.1*var_Per" "0"       "0"       "0"
## Cog "0"           "1"       "0.1*cor" "0"
## SO  "0"           "0.1*cor" "1"       "0"
## SC  "0"           "0"       "0"       "0.1*var_SC"

## Second stage analysis
fixed2 <- tssem2(fixed1, Amatrix=A1, Smatrix=S1, diag.constraints=TRUE,
                 intervals.type="LB")
## summary(fixed2)

## Rerun to clear the condition code
fixed2 <- rerun(fixed2, silent=TRUE)

## [1] "0.127853885947352,-0.26881276507009,-0.0589200100053669,-0.2749556988

summary(fixed2)

##
## Call:
## wls(Cov = pooledS, asyCov = tssem1.obj$acovS, n = sum(tssem1.obj$n),
##     Amatrix = Amatrix, Smatrix = Smatrix, Fmatrix = Fmatrix,
##     diag.constraints = diag.constraints, cor.analysis = cor.analysis,
##     intervals.type = intervals.type, mx.algebras = mx.algebras,
##     model.name = model.name, suppressWarnings = suppressWarnings,
##     silent = silent, run = run)
##
## 95% confidence intervals: Likelihood-based statistic
## Coefficients:
```

```
##           Estimate Std.Error  lbound  ubound z value Pr(>|z|)
## Cog2Per    0.1279        NA  0.0315  0.2252      NA       NA
## SO2Per    -0.0589        NA -0.1543  0.0366      NA       NA
## SC2Per     0.3999        NA  0.3158  0.4848      NA       NA
## Cog2SC    -0.2688        NA -0.3537 -0.1845      NA       NA
## SO2SC     -0.2750        NA -0.3597 -0.1907      NA       NA
## var_Per    0.8509        NA  0.7896  0.9018      NA       NA
## cor        0.5262        NA  0.4670  0.5855      NA       NA
## var_SC     0.7743        NA  0.7093  0.8316      NA       NA
##
## Goodness-of-fit indices:
##                                              Value
## Sample size                                    633
## Chi-square of target model                       0
## DF of target model                               0
## p value of target model                          0
## Number of constraints imposed on "Smatrix"       2
## DF manually adjusted                             0
## Chi-square of independence model               523
## DF of independence model                         6
## RMSEA                                            0
## SRMR                                             0
## TLI                                           -Inf
## CFI                                              1
## AIC                                              0
## BIC                                              0
## OpenMx status1: 0 ("0" or "1": The optimization is considered fine.
## Other values indicate problems.)
```

5.2.2 *Fixed-effects model with subgroup analysis*

Stage 1 analysis The above analysis indicates that the correlation matrices are very heterogeneous. This section illustrates how to group the studies into groups. If the studies become homogeneous, the grouping variable may be used to explain the heterogeneity.

```
## Display Type of sport
Becker09$Type_of_sport

##  [1] "Individual" "Individual" "Team"       "Individual" "Individual"
##  [6] "Individual" "Team"       "Team"       "Team"       "Individual"

cluster1 <- tssem1(Becker09$data, Becker09$n, method="FEM",
                   cluster=Becker09$Type_of_sport)
summary(cluster1)

## $Individual
##
## Call:
## tssem1FEM(my.df = data.cluster[[i]], n = n.cluster[[i]], cor.analysis = co
##     model.name = model.name, suppressWarnings = suppressWarnings)
```

```
##
## Coefficients:
##        Estimate Std.Error z value Pr(>|z|)
## S[1,2]  -0.1283    0.0561   -2.29    0.022 *
## S[1,3]  -0.2124    0.0545   -3.90  9.7e-05 ***
## S[1,4]   0.4900    0.0439   11.17  < 2e-16 ***
## S[2,3]   0.4732    0.0436   10.84  < 2e-16 ***
## S[2,4]  -0.3852    0.0476   -8.08  6.7e-16 ***
## S[3,4]  -0.4663    0.0439  -10.63  < 2e-16 ***
## ---
## Signif. codes:  0 '***' 0.001 '**' 0.01 '*' 0.05 '.' 0.1 ' ' 1
##
## Goodness-of-fit indices:
##                                   Value
## Sample size                      368.00
## Chi-square of target model       134.35
## DF of target model                25.00
## p value of target model            0.00
## Chi-square of independence model 396.83
## DF of independence model          31.00
## RMSEA                              0.27
## SRMR                               0.22
## TLI                                0.63
## CFI                                0.70
## AIC                               84.35
## BIC                              -13.35
## OpenMx status1: 0 ("0" or "1": The optimization is considered fine.
## Other values indicate problems.)
##
## $Team
##
## Call:
## tssem1FEM(my.df = data.cluster[[i]], n = n.cluster[[i]], cor.analysis = co
##     model.name = model.name, suppressWarnings = suppressWarnings)
##
## Coefficients:
##        Estimate Std.Error z value Pr(>|z|)
## S[1,2]  0.00476   0.06373    0.07  0.94051
## S[1,3] -0.08784   0.06273   -1.40  0.16146
## S[1,4]  0.20922   0.06129    3.41  0.00064 ***
## S[2,3]  0.58469   0.04081   14.33  < 2e-16 ***
## S[2,4] -0.44612   0.05172   -8.63  < 2e-16 ***
## S[3,4] -0.34642   0.05648   -6.13  8.6e-10 ***
## ---
## Signif. codes:  0 '***' 0.001 '**' 0.01 '*' 0.05 '.' 0.1 ' ' 1
##
## Goodness-of-fit indices:
##                                   Value
## Sample size                      265.00
## Chi-square of target model        49.88
## DF of target model                15.00
```

```
## p value of target model                  0.00
## Chi-square of independence model 231.94
## DF of independence model                21.00
## RMSEA                                     0.19
## SRMR                                      0.15
## TLI                                       0.77
## CFI                                       0.83
## AIC                                      19.88
## BIC                                     -33.81
## OpenMx status1: 0 ("0" or "1": The optimization is considered fine.
## Other values indicate problems.)
```

The *LR* statistics and the goodness-of-fit indicate that the correlation matrices are still heterogeneous. Grouping the studies does not help.

Stage 2 analysis As an illustration, we still show how to conduct the stage two analysis though we are not going to interpret the results.

```
## Second stage analysis
cluster2 <- tssem2(cluster1, Amatrix=A1, Smatrix=S1, diag.constraints=TRUE,
                   intervals.type="LB")
summary(cluster2)

## $Individual
##
## Call:
## wls(Cov = pooledS, asyCov = tssem1.obj$acovS, n = sum(tssem1.obj$n),
##     Amatrix = Amatrix, Smatrix = Smatrix, Fmatrix = Fmatrix,
##     diag.constraints = diag.constraints, cor.analysis = cor.analysis,
##     intervals.type = intervals.type, mx.algebras = mx.algebras,
##     model.name = model.name, suppressWarnings = suppressWarnings,
##     silent = silent, run = run)
##
## 95% confidence intervals: Likelihood-based statistic
## Coefficients:
##          Estimate Std.Error   lbound   ubound z value Pr(>|z|)
## Cog2Per   0.07346        NA -0.04522  0.19328      NA       NA
## SO2Per   -0.00703        NA -0.12900  0.11555      NA       NA
## SC2Per    0.51507        NA  0.41275  0.61958      NA       NA
## Cog2SC   -0.21202        NA -0.31951 -0.10507      NA       NA
## SO2SC    -0.36595        NA -0.46998 -0.26281      NA       NA
## var_Per   0.75552        NA  0.66351  0.83292      NA       NA
## cor       0.47317        NA  0.38759  0.55875      NA       NA
## var_SC    0.74770        NA  0.65891  0.82392      NA       NA
##
## Goodness-of-fit indices:
##                                              Value
## Sample size                                    368
## Chi-square of target model                       0
## DF of target model                               0
## p value of target model                          0
```

```
## Number of constraints imposed on "Smatrix"     2
## DF manually adjusted                           0
## Chi-square of independence model             340
## DF of independence model                       6
## RMSEA                                          0
## SRMR                                           0
## TLI                                         -Inf
## CFI                                            1
## AIC                                            0
## BIC                                            0
## OpenMx status1: 0 ("0" or "1": The optimization is considered fine.
## Other values indicate problems.)
##
## $Team
##
## Call:
## wls(Cov = pooledS, asyCov = tssem1.obj$acovS, n = sum(tssem1.obj$n),
##     Amatrix = Amatrix, Smatrix = Smatrix, Fmatrix = Fmatrix,
##     diag.constraints = diag.constraints, cor.analysis = cor.analysis,
##     intervals.type = intervals.type, mx.algebras = mx.algebras,
##     model.name = model.name, suppressWarnings = suppressWarnings,
##     silent = silent, run = run)
##
## 95% confidence intervals: Likelihood-based statistic
## Coefficients:
##         Estimate Std.Error   lbound   ubound z value Pr(>|z|)
## Cog2Per  0.17876        NA  0.01952  0.34118      NA       NA
## SO2Per  -0.10484        NA -0.25565  0.04528      NA       NA
## SC2Per   0.25265        NA  0.11707  0.38957      NA       NA
## Cog2SC  -0.37009        NA -0.50601 -0.23688      NA       NA
## SO2SC   -0.13003        NA -0.27022  0.00904      NA       NA
## var_Per  0.93708        NA  0.86343  0.98288      NA       NA
## cor      0.58469        NA  0.50465  0.66473      NA       NA
## var_SC   0.78985        NA  0.68858  0.87176      NA       NA
##
## Goodness-of-fit indices:
##                                            Value
## Sample size                                  265
## Chi-square of target model                     0
## DF of target model                             0
## p value of target model                        0
## Number of constraints imposed on "Smatrix"     2
## DF manually adjusted                           0
## Chi-square of independence model             288
## DF of independence model                       6
## RMSEA                                          0
## SRMR                                           0
## TLI                                         -Inf
## CFI                                            1
## AIC                                            0
## BIC                                            0
```

```
## OpenMx status1: 0 ("0" or "1": The optimization is considered fine.
## Other values indicate problems.)
```

Plot the figures When there is a cluster variable, it is of interest to display the differences on the parameter estimates. We may use the following code to plot the models for the individual sport and the team sport.

```
## Convert the model to semPlotModel object with 2 plots
## Use the short forms of the variable names to simplify the figures
my.plots <- lapply(X=cluster2, FUN=meta2semPlot,
                   manNames=c("Per","Cog","SO","SC") )

## Setup two plots
layout(t(1:2))
## The labels are overlapped. We may modify it by using layout="spring"
semPaths(my.plots[[1]], whatLabels="est", nCharNodes=10,
         color="orange", layout="spring", edge.label.cex=0.8)
title("Individual sport")

semPaths(my.plots[[2]], whatLabels="est", nCharNodes=10,
         color="skyblue", layout="spring", edge.label.cex=0.8)
title("Team sport")
```

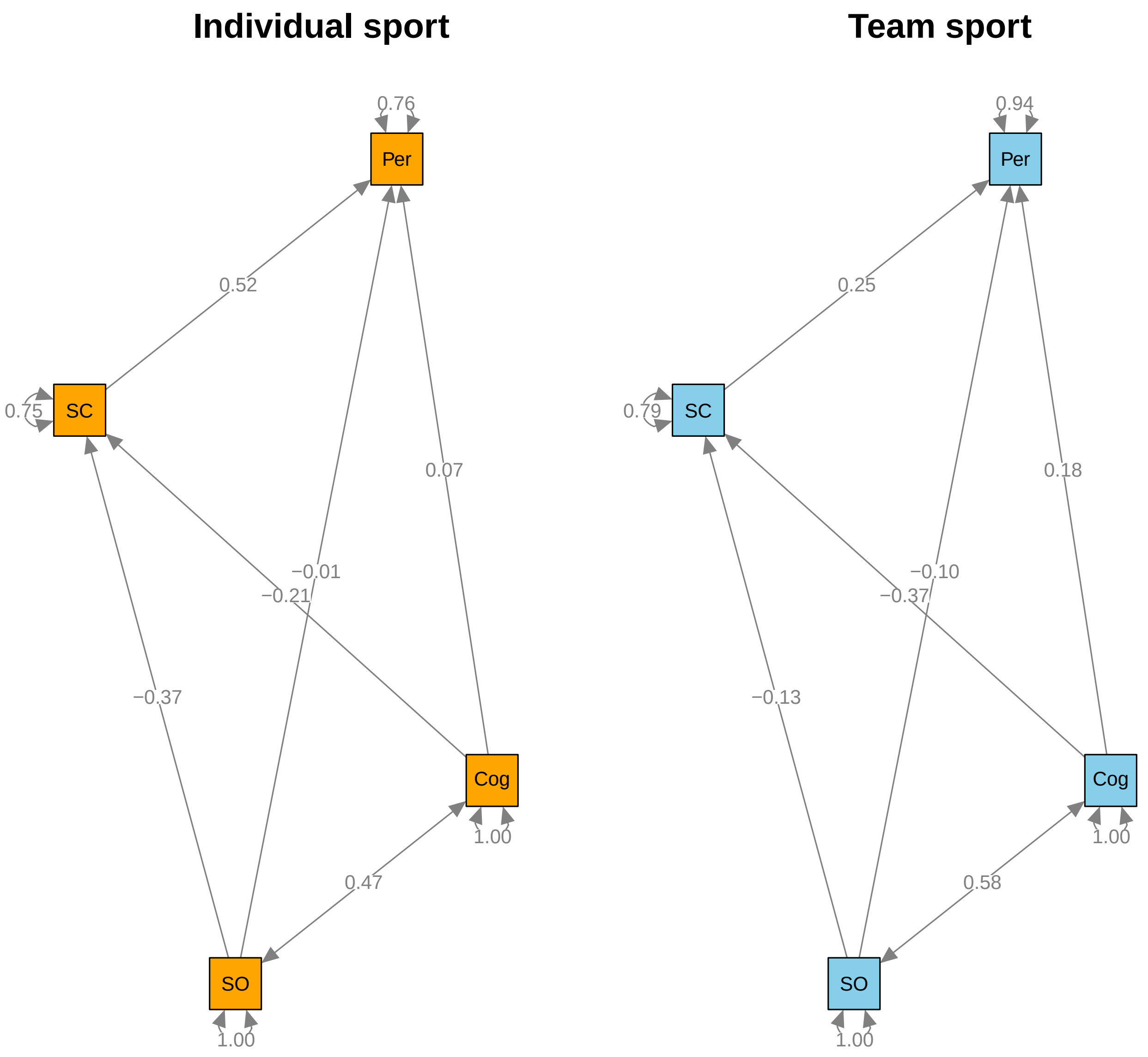

5.2.3 *Random-effects model*

Stage 1 analysis We may conduct a random-effects TSSEM with the following syntax. Since there is not enough data, we restrict the structure of the variance component of the random effects by specifying `RE.type="Diag"`. The I^2 of the correlation coefficients varies from 0.00 to 0.90. A random-effects model is more appropriate than a fixed-effects model for this data set.

```
## First stage analysis
random1 <- tssem1(Becker09$data, Becker09$n, method="REM",
                  RE.type="Diag")
summary(random1)


##
## Call:
## meta(y = ES, v = acovR, RE.constraints = Diag(x = paste(RE.startvalues,
##     "*Tau2_", 1:no.es, "_", 1:no.es, sep = "")), RE.lbound = RE.lbound,
##     I2 = I2, model.name = model.name, suppressWarnings = TRUE,
##     silent = silent, run = run)
##
## 95% confidence intervals: z statistic approximation
## Coefficients:
##              Estimate Std.Error    lbound    ubound z value Pr(>|z|)
## Intercept1 -7.28e-02  1.12e-01 -2.92e-01  1.46e-01   -0.65    0.515
## Intercept2 -1.60e-01  8.25e-02 -3.22e-01  1.66e-03   -1.94    0.052
## Intercept3  3.22e-01  8.10e-02  1.64e-01  4.81e-01    3.98    7e-05
## Intercept4  5.35e-01  2.95e-02  4.77e-01  5.93e-01   18.13   <2e-16
## Intercept5 -4.57e-01  4.34e-02 -5.43e-01 -3.72e-01  -10.54   <2e-16
## Intercept6 -4.64e-01  4.88e-02 -5.60e-01 -3.69e-01   -9.52   <2e-16
## Tau2_1_1    1.07e-01  5.35e-02  2.14e-03  2.12e-01    2.00    0.045
## Tau2_2_2    4.33e-02  2.70e-02 -9.65e-03  9.63e-02    1.60    0.109
## Tau2_3_3    3.63e-02  2.28e-02 -8.34e-03  8.08e-02    1.59    0.111
## Tau2_4_4    1.00e-10  3.38e-03 -6.63e-03  6.63e-03    0.00    1.000
## Tau2_5_5    5.57e-03  6.68e-03 -7.53e-03  1.87e-02    0.83    0.405
## Tau2_6_6    9.15e-03  8.04e-03 -6.61e-03  2.49e-02    1.14    0.255
##
## Intercept1
## Intercept2 .
## Intercept3 ***
## Intercept4 ***
## Intercept5 ***
## Intercept6 ***
## Tau2_1_1   *
## Tau2_2_2
## Tau2_3_3
## Tau2_4_4
## Tau2_5_5
## Tau2_6_6
## ---
## Signif. codes:  0 '***' 0.001 '**' 0.01 '*' 0.05 '.' 0.1 ' ' 1
##
## Q statistic on the homogeneity of effect sizes: 340.1
## Degrees of freedom of the Q statistic: 46
## P value of the Q statistic: 0
##
## Heterogeneity indices (based on the estimated Tau2):
##                              Estimate
## Intercept1: I2 (Q statistic)     0.90
## Intercept2: I2 (Q statistic)     0.76
```

```
## Intercept3: I2 (Q statistic)     0.76
## Intercept4: I2 (Q statistic)     0.00
## Intercept5: I2 (Q statistic)     0.34
## Intercept6: I2 (Q statistic)     0.46
##
## Number of studies (or clusters): 10
## Number of observed statistics: 52
## Number of estimated parameters: 12
## Degrees of freedom: 40
## -2 log likelihood: -30.37
## OpenMx status1: 0 ("0" or "1": The optimization is considered fine.
## Other values indicate problems.)
```

Stage 2 analysis Since the model is a saturated model, the *LR* statistic is 0 with 0 *df*. When there are mediators, we may also want to estimate the indirect effects. The `tssem2()` function allows us to include arbitrary algebras. For example, we may define the indirect effects via `Cog` and `SO` separately and totally with the following syntax. LBCI on these values may also be obtained. The results show that the indirect effects via `Cog` and `SO` separately and totally are -0.1075, -0.1099 and -0.2174, respectively. All of these effects are statistically significant.

```
## Second stage analysis
random2 <- tssem2(fixed1, Amatrix=A1, Smatrix=S1, diag.constraints=TRUE,
                  intervals.type="LB", model.name="TSSEM2 Becker09",
                  mx.algebras=list( Cog=mxAlgebra(Cog2SC*SC2Per, name="Cog"),
                                    SO=mxAlgebra(SO2SC*SC2Per, name="SO"),
                                    Cog_SO=mxAlgebra(Cog2SC*SC2Per+SO2SC*SC2P
                                    name="Cog_SO")) )
summary(random2)

##
## Call:
## wls(Cov = pooledS, asyCov = tssem1.obj$acovS, n = sum(tssem1.obj$n),
##     Amatrix = Amatrix, Smatrix = Smatrix, Fmatrix = Fmatrix,
##     diag.constraints = diag.constraints, cor.analysis = cor.analysis,
##     intervals.type = intervals.type, mx.algebras = mx.algebras,
##     model.name = model.name, suppressWarnings = suppressWarnings,
##     silent = silent, run = run)
##
## 95% confidence intervals: Likelihood-based statistic
## Coefficients:
##          Estimate Std.Error  lbound  ubound z value Pr(>|z|)
## Cog2Per    0.1279        NA  0.0315  0.2252      NA       NA
## SO2Per    -0.0589        NA -0.1543  0.0366      NA       NA
## SC2Per     0.3999        NA  0.3158  0.4848      NA       NA
## Cog2SC    -0.2688        NA -0.3537 -0.1845      NA       NA
## SO2SC     -0.2750        NA -0.3597 -0.1907      NA       NA
## var_Per    0.8509        NA  0.7896  0.9018      NA       NA
## cor        0.5262        NA  0.4670  0.5855      NA       NA
## var_SC     0.7743        NA  0.7093  0.8316      NA       NA
##
```

```
## mxAlgebras objects (and their 95% likelihood-based CIs):
##                lbound Estimate   ubound
## Cog[1,1]     -0.1528  -0.1075 -0.07005
## SO[1,1]      -0.1558  -0.1099 -0.07215
## Cog_SO[1,1] -0.2781  -0.2174 -0.16630
##
## Goodness-of-fit indices:
##                                             Value
## Sample size                                   633
## Chi-square of target model                      0
## DF of target model                              0
## p value of target model                         0
## Number of constraints imposed on "Smatrix"      2
## DF manually adjusted                            0
## Chi-square of independence model              523
## DF of independence model                        6
## RMSEA                                           0
## SRMR                                            0
## TLI                                          -Inf
## CFI                                             1
## AIC                                             0
## BIC                                             0
## OpenMx status1: 6 ("0" or "1": The optimization is considered fine.
## Other values indicate problems.)
```

We may plot the model and label the parameters for checking.

```
## Convert the model to semPlotModel object
my.plot <- meta2semPlot(random2, manNames=c("Per","Cog","SO","SC") )

## Plot the model with labels
## The labels are overlapped. We may modify it by using layout="spring"
semPaths(my.plot, whatLabels="path", nCharEdges=10,  nCharNodes=10,
         layout="spring", color="yellow", edge.label.cex=0.8)
```

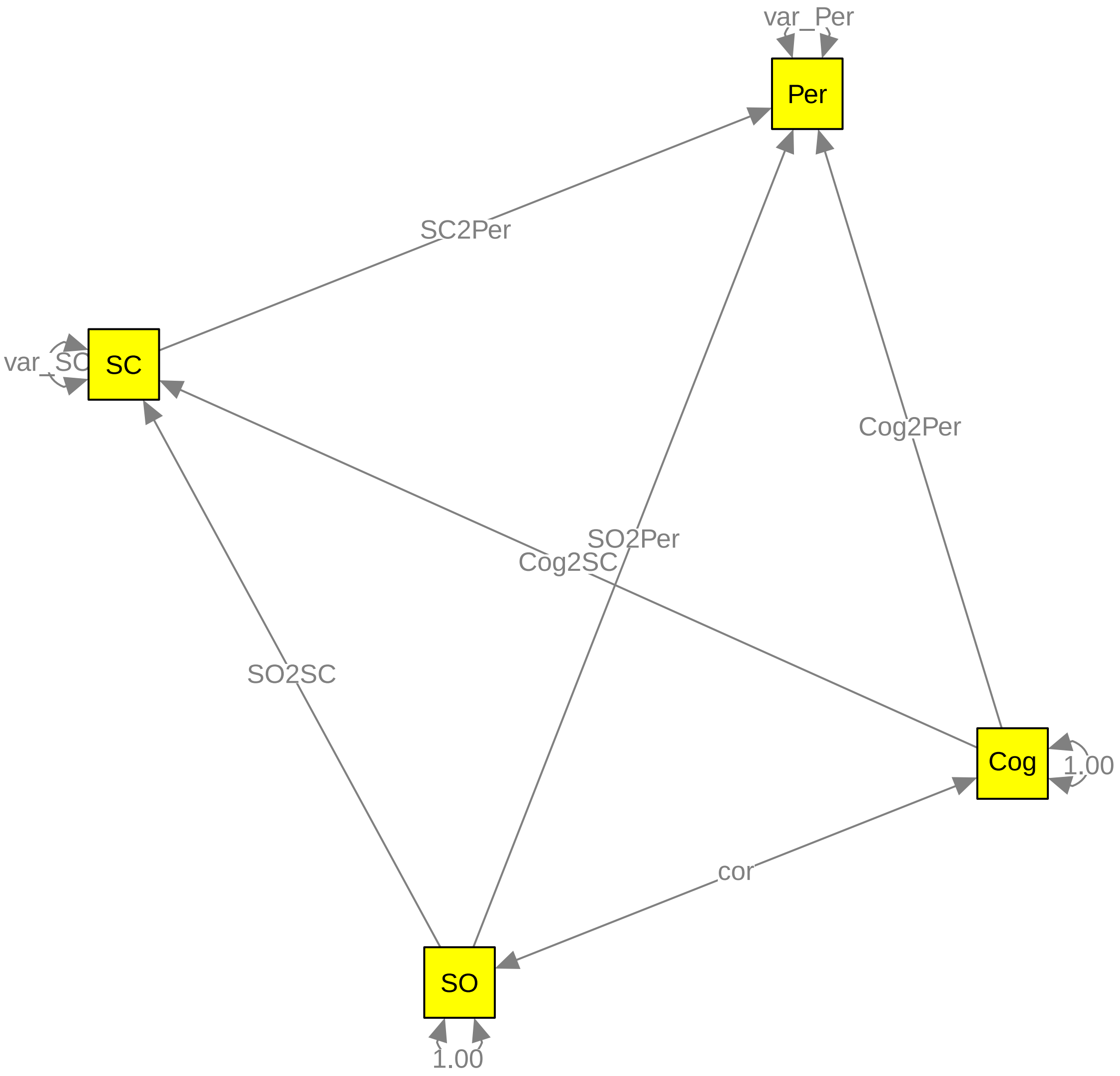

Figure 4. Plot of parameter labels

We may also plot the parameter estimates in the figure.

```
## Plot the parameter estimates
semPaths(my.plot, whatLabels="est", nCharNodes=10, layout="spring",
         color="green", edge.label.cex=1.2)
```

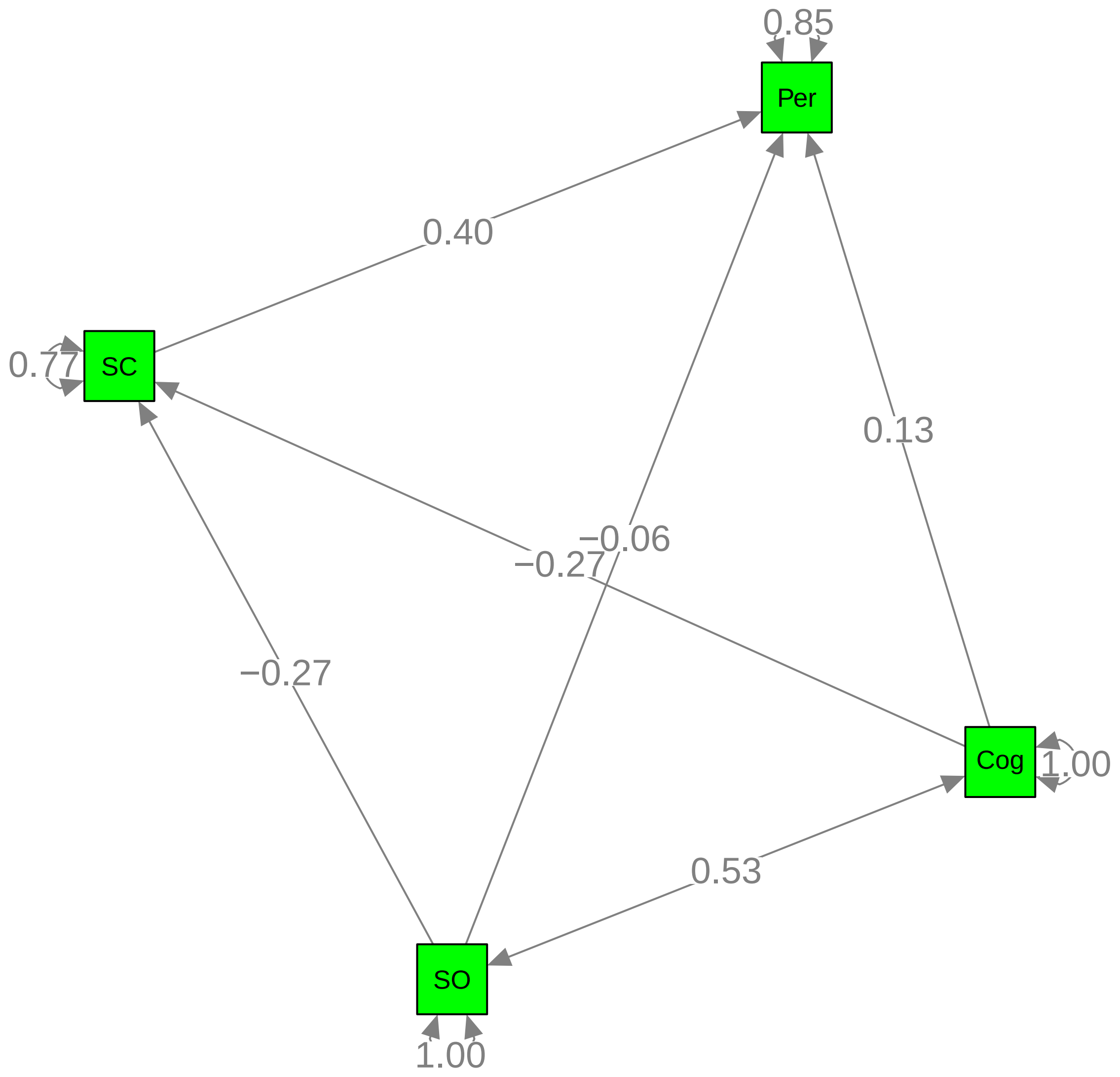

Figure 5. Plot of parameter labels

The above analyses were conducted based on the following R Packages.

```
sessionInfo()

## R version 3.1.1 (2014-07-10)
## Platform: i686-pc-linux-gnu (32-bit)
```

```
##
## locale:
##  [1] LC_CTYPE=en_SG.UTF-8       LC_NUMERIC=C
##  [3] LC_TIME=en_SG.UTF-8        LC_COLLATE=en_SG.UTF-8
##  [5] LC_MONETARY=en_SG.UTF-8    LC_MESSAGES=en_SG.UTF-8
##  [7] LC_PAPER=en_SG.UTF-8       LC_NAME=C
##  [9] LC_ADDRESS=C               LC_TELEPHONE=C
## [11] LC_MEASUREMENT=en_SG.UTF-8 LC_IDENTIFICATION=C
##
## attached base packages:
## [1] parallel  stats     graphics  grDevices utils     datasets
## [7] methods   base
##
## other attached packages:
## [1] semPlot_1.0.1     metafor_1.9-4     Matrix_1.1-4
## [4] Formula_1.1-2     metaSEM_0.9-0     OpenMx_2.0.0-4004
## [7] MASS_7.3-35       digest_0.6.4      knitr_1.8
##
## loaded via a namespace (and not attached):
##  [1] acepack_1.3-3.3     car_2.0-22          cluster_1.15.3
##  [4] colorspace_1.2-4    corpcor_1.6.7       ellipse_0.3-8
##  [7] evaluate_0.5.5      foreign_0.8-61      formatR_1.0
## [10] ggplot2_1.0.0       glasso_1.8          grid_3.1.1
## [13] gtable_0.1.2        highr_0.4           Hmisc_3.14-5
## [16] huge_1.2.6          igraph_0.7.1        jpeg_0.1-8
## [19] lattice_0.20-29     latticeExtra_0.6-26 lavaan_0.5-17.698
## [22] lisrelToR_0.1.4     lme4_1.1-7          matrixcalc_1.0-3
## [25] minqa_1.2.4         mnormt_1.5-1        munsell_0.4.2
## [28] nlme_3.1-118        nloptr_1.0.4        nnet_7.3-8
## [31] pbivnorm_0.5-1      plyr_1.8.1          png_0.1-7
## [34] proto_0.3-10        psych_1.4.8.11      qgraph_1.2.5
## [37] quadprog_1.5-5      RColorBrewer_1.0-5  Rcpp_0.11.3
## [40] reshape2_1.4        rockchalk_1.8.86    rpart_4.1-8
## [43] scales_0.2.4        sem_3.1-5           sna_2.3-2
## [46] splines_3.1.1       stats4_3.1.1        stringr_0.6.2
## [49] survival_2.37-7     tools_3.1.1         XML_3.98-1.1
```

REFERENCES

Aloe, A. M., Amo, L. C., and Shanahan, M. E. (2014), Classroom management self-efficacy and burnout: A multivariate meta-analysis, *Educational Psychology Review*, 26, 1, 101–126, doi:10.1007/s10648-013-9244-0

Becker, B. J. (1983), Influence again: A comparison of methods for meta-analysis, in Paper presented at the annual meeting of the American Educational Research Association (Montreal)

Becker, B. J. (1992), Using results from replicated studies to estimate linear models, *Journal of Educational Statistics*, 17, 4, 341–362, doi:10.3102/10769986017004341

Becker, B. J. (2009), Model-based meta-analysis, in H. Cooper, L. V. Hedges, and J. C. Valentine, eds., The handbook of research synthesis and meta-analysis (Russell Sage Foundation, New York), 2nd edition, 377–395

Berkey, C. S., Hoaglin, D. C., Antczak-Bouckoms, A., Mosteller, F., and Colditz, G. A. (1998), Meta-analysis of multiple outcomes by regression with random effects, *Statistics in Medicine*, 17, 22, 2537–2550, doi:10.1002/(SICI)1097-0258(19981130)17:22⟨2537::AID-SIM953⟩3.0.CO;2-C

Cheung, M. W.-L. (2009), Constructing approximate confidence intervals for parameters with structural equation models, *Structural Equation Modeling: A Multidisciplinary Journal*, 16, 2, 267–294, doi:10.1080/10705510902751291

Cheung, M. W.-L. (2013), Multivariate meta-analysis as structural equation models, *Structural Equation Modeling: A Multidisciplinary Journal*, 20, 3, 429–454, doi:10.1080/10705511.2013.797827

Cheung, M. W.-L. (2014), Fixed- and random-effects meta-aanalytic structural equation modeling: Examples and analyses in R, *Behavior Research Methods*, 46, 29–40, doi:10.3758/s13428-013-0361-y

Cheung, M. W.-L. (2015), Meta-analysis: A structural equation modeling approach (John Wiley & Sons, Ltd), in press

Cheung, M. W.-L. and Chan, W. (2005), Classifying correlation matrices into relatively homogeneous subgroups: A cluster analytic approach, *Educational and Psychological Measurement*, 65, 6, 954–979, doi:10.1177/0013164404273946

Cooper, H., Valentine, J. C., Charlton, K., and Melson, A. (2003), The effects of modified school calendars on student achievement and on school and community attitudes, *Review of Educational Research*, 73, 1, 1 –52, doi:10.3102/00346543073001001

Craft, L. L., Magyar, T. M., Becker, B. J., and Feltz, D. L. (2003), The relationship between the competitive state anxiety inventory-2 and sport performance: a meta-analysis, *Journal of Sport and Exercise Psychology*, 25, 1, 44–65

Digman, J. M. (1997), Higher-order factors of the big five, *Journal of Personality and Social Psychology*, 73, 6, 1246–1256, doi:10.1037/0022-3514.73.6.1246

Jaramillo, F., Mulki, J. P., and Marshall, G. W. (2005), A meta-analysis of the relationship between organizational commitment and salesperson job performance: 25 years of research, *Journal of Business Research*, 58, 6, 705–714, doi:10.1016/j.jbusres.2003.10.004

Konstantopoulos, S. (2011), Fixed effects and variance components estimation in three-level meta-analysis, *Research Synthesis Methods*, 2, 1, 61–76, doi:10.1002/jrsm.35

McArdle, J. J. and McDonald, R. P. (1984), Some algebraic properties of the reticular action model for moment structures, *British Journal of Mathematical and Statistical Psychology*, 37, 2, 234–251, doi:10.1111/j.2044-8317.1984.tb00802.x

Neale, M. C. and Miller, M. B. (1997), The use of likelihood-based confidence intervals in genetic models, *Behavior Genetics*, 27, 2, 113–120, doi:10.1023/A:1025681223921

Stoel, R. D., Garre, F. G., Dolan, C., and van den Wittenboer, G. (2006), On the likelihood ratio test in structural equation modeling when parameters are subject to boundary constraints, *Psychological Methods*, 11, 4, 439–455, doi:10.1037/1082-989X.11.4.439
